# Functional connectivity MRI quality control procedures in CONN

**DOI:** 10.3389/fnins.2023.1092125

**Published:** 2023-03-23

**Authors:** Francesca Morfini, Susan Whitfield-Gabrieli, Alfonso Nieto-Castañón

**Affiliations:** ^1^Department of Psychology, Northeastern University, Boston, MA, United States; ^2^Athinoula A. Martinos Center for Biomedical Imaging, Massachusetts General Hospital and Harvard Medical School, Boston, MA, United States; ^3^Department of Brain and Cognitive Sciences and McGovern Institute for Brain Research, Massachusetts Institute of Technology, Cambridge, MA, United States; ^4^McGovern Institute for Brain Research, Massachusetts Institute of Technology, Cambridge, MA, United States; ^5^Department of Speech, Language, and Hearing Sciences, Boston University, Boston, MA, United States

**Keywords:** fMRI, quality control, neuroimaging (anatomic), CONN toolbox, functional connectivity, resting state, preprocessing, denoising

## Abstract

Quality control (QC) for functional connectivity magnetic resonance imaging (FC-MRI) is critical to ensure the validity of neuroimaging studies. Noise confounds are common in MRI data and, if not accounted for, may introduce biases in functional measures affecting the validity, replicability, and interpretation of FC-MRI study results. Although FC-MRI analysis rests on the assumption of adequate data processing, QC is underutilized and not systematically reported. Here, we describe a quality control pipeline for the visual and automated evaluation of MRI data implemented as part of the CONN toolbox. We analyzed publicly available resting state MRI data (*N* = 139 from 7 MRI sites) from the FMRI Open QC Project. Preprocessing steps included realignment, unwarp, normalization, segmentation, outlier identification, and smoothing. Data denoising was performed based on the combination of scrubbing, motion regression, and aCompCor – a principal component characterization of noise from minimally eroded masks of white matter and of cerebrospinal fluid tissues. Participant-level QC procedures included visual inspection of raw-level data and of representative images after each preprocessing step for each run, as well as the computation of automated descriptive QC measures such as average framewise displacement, average global signal change, prevalence of outlier scans, MNI to anatomical and functional overlap, anatomical to functional overlap, residual BOLD timeseries variability, effective degrees of freedom, and global correlation strength. Dataset-level QC procedures included the evaluation of inter-subject variability in the distributions of edge connectivity in a 1,000-node graph (FC distribution displays), and the estimation of residual associations across participants between functional connectivity strength and potential noise indicators such as participant’s head motion and prevalence of outlier scans (QC-FC analyses). QC procedures are demonstrated on the reference dataset with an emphasis on visualization, and general recommendations for best practices are discussed in the context of functional connectivity and other fMRI analysis. We hope this work contributes toward the dissemination and standardization of QC testing performance reporting among peers and in scientific journals.

## 1. Introduction

Since its inception, neuroimaging has escalated our understanding of the brain in both health and disease. Functional magnetic resonance imaging (fMRI) is among the most common neuroimaging techniques, as it allows us to approximate neural activity *in vivo* and non-invasively by measuring the blood oxygenation level-dependent (BOLD) signal. Brain functional connectivity (FC), or the temporal coupling of BOLD signals from anatomically distant regions, is widely used to probe neural functioning, neurodiversity, and their relationship with behavior during explicit or implicit (i.e., at rest) tasks. However, the BOLD signal is noisy and only marginally representative of neural activity. It is generated from complex interactions between neuronal, metabolic, cardiac, vigilance, and other physiological processes (Bianciardi et al., 2009; Liu, 2016; Liu and Falahpour, 2020) and is commonly affected by machine-related and participant-specific characteristics. In many fMRI analyses, these noise sources act as nuisance effects, increasing variability of the BOLD signal and ultimately reducing the power and replicability of fMRI analysis results. In functional connectivity analyses, their effect is considerably more damaging, as many of these noise sources are highly correlated across different areas and will bias functional connectivity estimates, acting as confounder effects and affecting the validity and interpretation of FC-MRI analysis results.

Commonly, anatomical and functional data undergo a series of transformations aimed at minimizing the effects of these well-known sources of BOLD signal variability prior to statistical analysis. Functional and anatomical data are usually first preprocessed with a set of steps addressing mainly spatial properties of the data that are a direct consequence of the specificities of the fMRI acquisition procedure. Specifically, preprocessing focuses on intra-participant coregistration, e.g., compensating for head motion across different functional scans, correcting for inter-slice temporal differences and magnetic susceptibility distortions, when appropriate, as well as inter-participant coregistration, e.g., by spatially projecting each subject’s anatomy to a common reference space. However, despite these common preprocessing steps, functional timeseries after preprocessing usually still contain substantial variability associated with non-neural sources, including cardiac, respiratory, and residual subject motion effects, limiting the ability to effectively use these data for statistical analyses without additional control or correction of these factors. For these reasons, and particularly in the context of functional connectivity analyses, preprocessed functional timeseries are often usually then denoised by a combination of band-pass filtering and regression of temporal components characterizing these additional noise sources. Many effective alternatives have been suggested to achieve optimal preprocessing (Friston et al., 1996; Murphy et al., 2009; Chai et al., 2012; Hallquist et al., 2013; Power et al., 2014; Ciric et al., 2017) and denoising performance (Parkes et al., 2018; Maknojia et al., 2019; Tong et al., 2019; De Blasi et al., 2020; Golestani and Chen, 2022; for a review, see Caballero-Gaudes and Reynolds, 2017). Regardless of the specific pipelines applied, preprocessing and denoising have been shown to successfully reduce the effect of known nuisance factors.

However, the beneficial effect of preprocessing and denoising depends on the ability of each step to successfully achieve its intended goal. Quality control (QC) procedures are designed to evaluate the quality of the data and to detect potential problems either in the original data or arising from failed or insufficient preprocessing and denoising steps. Quality control is an integral part of preparing fMRI data for statistical analyses, as without it there is no meaningful way to avoid problems in the data from affecting statistical analyses, leading to results that may fail to replicate, may be disproportionately influenced by the presence of outliers, or may be confounded by physiological or other non-neural sources of variability among participants. While data quality is an agreed-upon essential element for fMRI analysis, what constitutes “good” data and “appropriate” QC procedures are still open questions. Perhaps owing to the complexity of assessing data quality in the absence of a ground truth, QC is often underappreciated and not systematically reported. Yet, QC and QC reporting are crucial to data interpretation and needed to develop standardized guidelines (Taylor et al., 2022).

Several studies have addressed the topic of MRI data quality, whether from the perspective of quality assurance (QA) or from a QC point of view. Although interwoven, QA and QC are complementary in that QA is usually a process-oriented approach aimed at preventing issues (e.g., Friedman and Glover, 2006; Glover et al., 2012; Liu et al., 2015; for a review see Lu et al., 2019), whereas QC is output-oriented and evaluates the quality of the images resulting from said process. As such, even an optimal QA does not address the objectives of QC testing. Recent efforts from the field have resulted in the proliferation of QC tools and protocols for the evaluation of specific analytical step (Backhausen et al., 2016; Storelli et al., 2019; Benhajali et al., 2020), pipelines-specific outputs (Griffanti et al., 2017; Raamana et al., 2020; Chou et al., 2022), and raw-level data [e.g., MRIQC (Esteban et al., 2017) and pyfMRIqc (Williams and Lindner, 2020)]. Additionally, many pipelines have been developed to preprocess (e.g., fMRIprep; Esteban et al., 2019), denoise (e.g., Tedana; DuPre et al., 2021), or generally analyze fMRI data from specific consortia [e.g., ABCD (Hagler et al., 2019), UK Biobank (Alfaro-Almagro et al., 2018), HCP (Marcus et al., 2013), Configurable Pipeline for the Analysis of Connectomes C-PAC^1^ (Craddock et al., 2013; Sikka et al., 2014)]. While principally focused on data analysis, these tools also strongly support automatic and visual QC, and effectively aid the identification of issues in the data and during data analysis. These works, together with our and the other papers presented in this special issue (Taylor et al., 2022), help build a rich diversity of approaches and perspectives. Each provides unique contributions which help expand the field and build a consensus on best practices.

In this study, we describe the quality control pipeline for volume-based connectivity analysis using the CONN toolbox (Whitfield-Gabrieli and Nieto-Castanon, 2012; Nieto-Castanon, 2020). We analyzed publicly available resting-state data (*n* = 139) from the FMRI Open QC Project (Taylor et al., 2022) to demonstrate participant-level and group-level QC procedures in an integrated framework with data preprocessing and denoising. Visual and automated QC procedures were demonstrated for the assessment of raw-level, preprocessed, and denoised data. Finally, we proposed a QC workflow based on the combination of visual and automated QC measures. Ultimately, we hope this work contributes toward the dissemination and standardization of QC testing and reporting.

## 2. Materials

### 2.1. Dataset overview

We analyzed data from the FMRI Open QC Project (Taylor et al., 2022) fmri-open-qc-rest collection v1.0.0, which combined subsamples of public data-packages including ABIDE and ABIDE-II (Di Martino et al., 2013), the Functional Connectome Project (Biswal et al., 2010), and OpenNeuro (Markiewicz et al., 2021). Data was accessed as already transformed nifti and json files curated to be in BIDS format v1.6.0 (Gorgolewski et al., 2016).

The fmri-open-qc-rest collection included (f)MRI data from 139 participants acquired with 3.0T MRI scanners from seven sites. Each participant had available data corresponding to one MRI scanning session when one anatomical image and one or two echo-planar imaging (EPI) resting state functional BOLD runs were collected.

### 2.2. Software information

MRI data processing and statistical analyses were performed using the CONN toolbox (RRID:SCR_009550) version 22.a (Nieto-Castanon and Whitfield-Gabrieli, 2022) and SPM version 12 release 7,771 (Wellcome Department of Imaging Neuroscience, UCL, London, United Kingdom) in MATLAB R2022a (The MathWorks Inc., Natick, MA, United Kingdom).

## 3. Methods

Code and scripts required to replicate the analysis presented in this manuscript can be found at https://github.com/alfnie/conn.

### 3.1. Preprocessing

**Figure 1:**
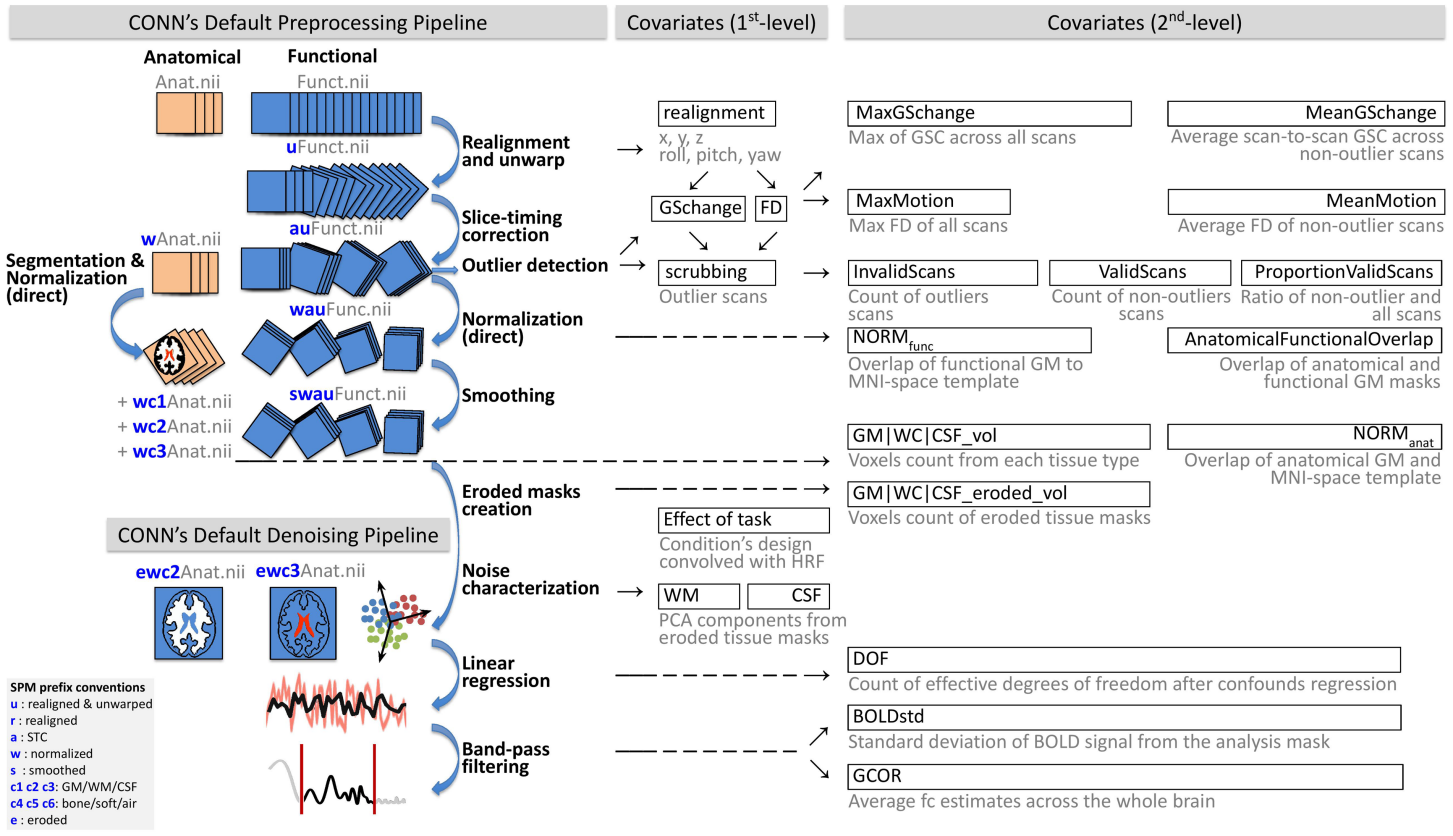
Schematic of preprocessing and denoising analysis flow and automated QC measures. The figure illustrates the CONN’s minimal default preprocessing and denoising pipelines and the automated quality control measures generated from each step. Automated QC measures were considered *Covariates (1st-level)* if they represented run-specific timeseries (i.e., one value per scan) or *Covariates (2nd-level)* if they were the collection of aggregated within-run estimates (i.e., one value per run). BOLD, blood oxygen level dependent; CSF, cerebrospinal fluid; DOF, degrees of freedom; FD, framewise displacement; GM, gray matter; GCOR, global correlation; GS, global signal; HRF, hemodynamic response function; QC, quality control; WM, white matter. This figure was adapted with permission from Nieto-Castanon (2020), Copyright^©^ 2020 Alfonso Nieto-Castanon.

Functional and anatomical images were preprocessed using the default minimal preprocessing pipeline in CONN (Nieto-Castanon, 2020, 2022), represented in Figure 1 (top). This pipeline includes functional *realignment and unwarp* (Andersson et al., 2001) for intermodality coregistration of all scans to the first scan, *slice-timing correction* (STC; Henson et al., 1999) compensating for acquisition time differences among different slices, *outlier detection* (Whitfield-Gabrieli et al., 2011) identifying individual scans with suprathreshold framewise displacement (FD) and/or global signal change (GSC) values, *direct functional normalization* (Calhoun et al., 2017) projecting functional images into standard Montreal Neurological Institute 152 (MNI) reference space resampled to 2 mm isotropic voxels, and spatial *smoothing* with a 8 mm full width at half maximum Gaussian kernel. Anatomical data preprocessing comprised *direct segmentation and normalization* (Ashburner and Friston, 2005) which iteratively performed tissue *segmentation* into six tissue classes, including gray matter (GM), white matter (WM), and cerebrospinal fluid (CSF) using SPM12 posterior tissue probability maps, and *normalization* to IXI-549 MNI space, resampling the output anatomical images to 1 mm isotropic voxels.

Several automated measures were extracted as run-level timeseries (i.e., as 1st-level covariates) at various stages of preprocessing, following Nieto-Castanon (2020, 2022). Table 1 (QC timeseries section) includes a summary of each of these QC timeseries definitions, and Figure 1 provides a schematic representation of all preprocessing steps and associated QC timeseries. The QC timeseries named realignment is estimated during the *realignment and unwarp* preprocessing step, and it represents the estimated participant in-scanner head motion. The individual parameters in this timeseries represent the degree of relative translation (three parameters, in mm units) and rotations (three parameters, in radians) of the head at each individual scan, when compared to its position at the beginning of the functional run. Following SPM12 convention, rotation parameters are defined using the real word-space point (coordinate 0,0,0) as the center of rotation. The QC timeseries named Global Signal Change (GSC) and Framewise Displacement (FD) are computed during the *outlier detection* preprocessing step. GSC timeseries are defined at each scan as the absolute value of the scan-to-scan change in global BOLD signal, using SPM global BOLD signal definition. GSC timeseries are then scaled to standard units within each run by subtracting their median value and dividing by 0.74 times their interquartile range (Whitfield-Gabrieli et al., 2011). FD timeseries are defined as the maximum change in the position of six points placed at the centers of each face in a 140 × 180 × 115 mm bounding box around the brain and undergoing the same rotations and translations as the participant’s head. From these measures, outlier scans are identified as the scans with FD values above 0.5 mm and/or GSC values above 3 standard deviations (Whitfield-Gabrieli et al., 2011), with the resulting list of potential outlier scans summarized in the QC timeseries named scrubbing.

**Table 1:** Summary of automated quality control measures.

QC timeseries (1st-level covariates)		
GSchange	The global signal change timeseries is computed as the absolute value of the scan-to-scan change in global BOLD signal, computed separately at each scan/timepoint and scaled to standard units within each run.	0 < x < ∞. Higher values indicate higher sudden variability in signal intensity.
FD	The framewise displacement timeseries is computed as the maximum change in the position of six control points placed at the center of a bounding box around the brain, computed separately at each scan/timepoint.	0 < x < ∞. Higher values indicate higher sudden displacements in head position.
Scrubbing	The scrubbing covariate contains one separate timeseries per identified outlier scan. Each of these timeseries contain a single 1-value at the identified scan, and 0-values at all other timepoints. They are computed by thresholding GSchange and FD at the desired values.	x ∈ {0,1}. 1 indicates a scan identified as a potential outlier
Realignment	The realignment covariate contains six timeseries, three characterizing head translations along the x/y/z directions in mm units, and three characterizing rotations around the x/y/z axes in radians.	-∞ < x < ∞. Higher absolute values indicate larger relative motion between a scan compared to the first scan within the same run
WM	The WM covariate contains multiple timeseries, characterizing the principal components of the BOLD signal within white matter areas, sorted by decreasing variance.	-∞ < x < ∞. Higher absolute values indicate larger departures from the average BOLD signal within WM
CSF	The CSF covariate contains multiple timeseries, characterizing the principal components of the BOLD signal within cerebrospinal fluid tissue areas, sorted by decreasing variance.	-∞ < x < ∞. Higher absolute values indicate larger departures from the average BOLD signal within CSF
QC summary measures (2nd-level covariates)		
MaxMotion	The maximum of motion is the maximum value of the FD timeserie from each run, calculated considering all original scans.	0 < x < ∞. Higher values indicate more extreme motion spikes.
InvalidScans	Invalid scans is the number of scans identified as outliers during outlier detection based on scan-to-scan GS and framewise displacement change.	0 < x < total number of scans. Higher values indicate higher presence of potential outlier scans.
ValidScans	Valid scans is the number of valid or non-outlier scans.	0 < x < total number of scans. Lower values indicate fewer surviving scans.
PVS	The proportion of valid scans is the ratio between non-outlier scans to all scans, representing a normalized measure of valid scans in the presence of potential differences in scanning lengths.	0 < x < 1. Lower values indicate higher presence of potential outlier scans.
MeanGSchange	The mean global signal change is the mean value of GSchange timeseries, calculated by aggregating GSchange across non-outlier scans only.	-∞ < x < ∞. Higher values indicate higher residual variability in the global signal after scrubbing
MeanMotion	The mean motion is the mean value of the FD timeseries, calculated by aggregating FD across non-outlier scans only.	0 < x < ∞. Higher values indicate higher residual motion after scrubbing.
NORM_func_	The normalized space to functional accuracy is the Dice similarity coefficient between the IXI-549 MNI-space gray matter tissue mask thresholded at a 25% probability level and the binarized GM masks derived from the functional data and thresholded at a level that produced the same number of suprathreshold voxels as in the MNI-space mask.	0 < x < 1. Lower values indicate a worse normalization of functional data.
NORM_anat_	The normalized space to anatomical accuracy is calculated similarly to NORM_func_ but it compares the IXI-549 gray matter mask to the binarized GM mask derived from the anatomical data instead.	0 < x < 1. Lower values represent worse normalization of anatomical data.
AFO	The anatomical-to-functional overlap is the Dice similarity coefficient between the anatomical gray matter mask, thresholded at a 50% probability level, and the functional gray matter mask, thresholded at a level that resulted in the same number of suprathreshold voxels.	0 < x < 1. Lower values represent a worse inter-modality coregistration.
tissue_vol	The gray matter, white matter, or cerebrospinal fluid tissue volumes is the count of voxels with tissue-specific probability >50% from participant-specific segmented anatomical tissue ROIs.	0 < x < ∞. Extreme values indicate a combination of individual anatomical differences and normalization performance.
tissue_eroded_vol	The tissue eroded volume is the count of voxels in the tissue-specific ROIS resulting from anatomical segmentation after a 1-voxel erosion procedure.	0 < x < ∞. Extreme values indicate a combination of individual anatomical differences and normalization performance.
DOF	The effective degrees of freedom are calculated as the total number of scans minus the number of regressors involved in the denoising’s linear regression step, multiplied by the fraction of the Nyquist frequency covered by denoising’s band-pass frequency filter.	-∞ < x < all original scans. Lower values indicate potential lack of precision when estimating modeled effects in the BOLD signal.
QC summary measures (2nd-level covariates)		
BOLDstd	The BOLD standard deviation is the temporal standard deviation of the BOLD signal, after grand-mean scaling to 100 across the entire brain and denoising, averaged across all runs and all voxels in the analysis mask.	0 < x < ∞. High values may indicate the presence of potential noise, while values close to 0 may indicate lack of retained signal.
GCOR	The mean global correlation (Saad et al., 2013) is the average of Pearson’s r correlation coefficients between the denoised BOLD timeseries of all pairs of voxels within the analysis mask.	-∞ < x < ∞. High absolute values may indicate the presence of residual noise sources in the BOLD signal.
QC-FC %	Quality Control to Functional Connectivity distributions (Ciric et al., 2017) represent the observed distribution of correlations across participants between individual QC measures and functional connectivity strength (edges in a fixed graph of 1,000 random voxels within the MNI-space gray matter template mask). QC-FC % match level represents the distance between these observed distributions and those that could be expected by chance, as computed using permutation analyses.	0% < x < 100%. Values above 95% indicate negligible modulations associated with nuisance factors in the correlation structure of the BOLD signal.

All quality control measures are automatically calculated by CONN (v22.a) during data preprocessing and denoising, but all could also be derived post-hoc from data fully or partially processed by other software.

In addition to being useful on their own to characterize image and subject properties during data acquisition in the scanner, relevant statistics of these 1st-level measures are also used to define additional summary measures, as shown in Table 1 (QC summary measures section) and discussed in section 3.3.2.

### 3.2. Denoising

In order to minimize the presence of non-neural noise sources, including cardiac, respiratory, and residual subject motion effects in the BOLD signal, functional data were denoised with the CONN fMRI default denoising pipeline (Nieto-Castanon, 2020). This pipeline comprises three main sequential steps (Figure 1, bottom) seeking to characterize noise components in the BOLD signal (*noise components extraction*) and minimize their effect on the BOLD timeseries (*linear regression* and *temporal band-pass filtering* steps). First, participant-specific minimally eroded WM and CSF masks were generated using a one-voxel binary 3D erosion of the corresponding tissue masks derived from each subject’s anatomical *segmentation*. The QC timeseries named WM and CSF (Table 1) are defined as the principal components of the BOLD signal extracted from these minimally eroded masks, following the anatomical aCompCor method (Behzadi et al., 2007), which has been shown to minimize the effect of nuisance confounds (Chai et al., 2012). Principal components from WM and CSF areas were computed after discounting motion and outlier effects (within a space orthogonal to the realignment and scrubbing QC timeseries).

Next, ordinary least squares regression removed from each voxel BOLD timeseries the effect of all identified noise components, including 5 components from white matter (from the QC timeseries WM), 5 components from CSF (from the QC timeseries CSF), 12 estimated participant-motion parameters (6 parameters from the QC timeseries realignment and their first order temporal derivatives), participant-specific outlier scans (from the QC timeseries scrubbing), as well as the effect of session and its first order derivative convolved with the canonical hemodynamic response function (aiming to minimize the influence of transients in the first few scans of each run), and constant and linear session effects (aiming to minimize the influence of linear trends in each run). Lastly, temporal band-pass filtering (0.008–0.09 HZ) was applied to each run individually (Hallquist et al., 2013) in order to focus on slowly varying BOLD signal fluctuations.

### 3.3. CONN quality control pipeline

**Figure 2:**
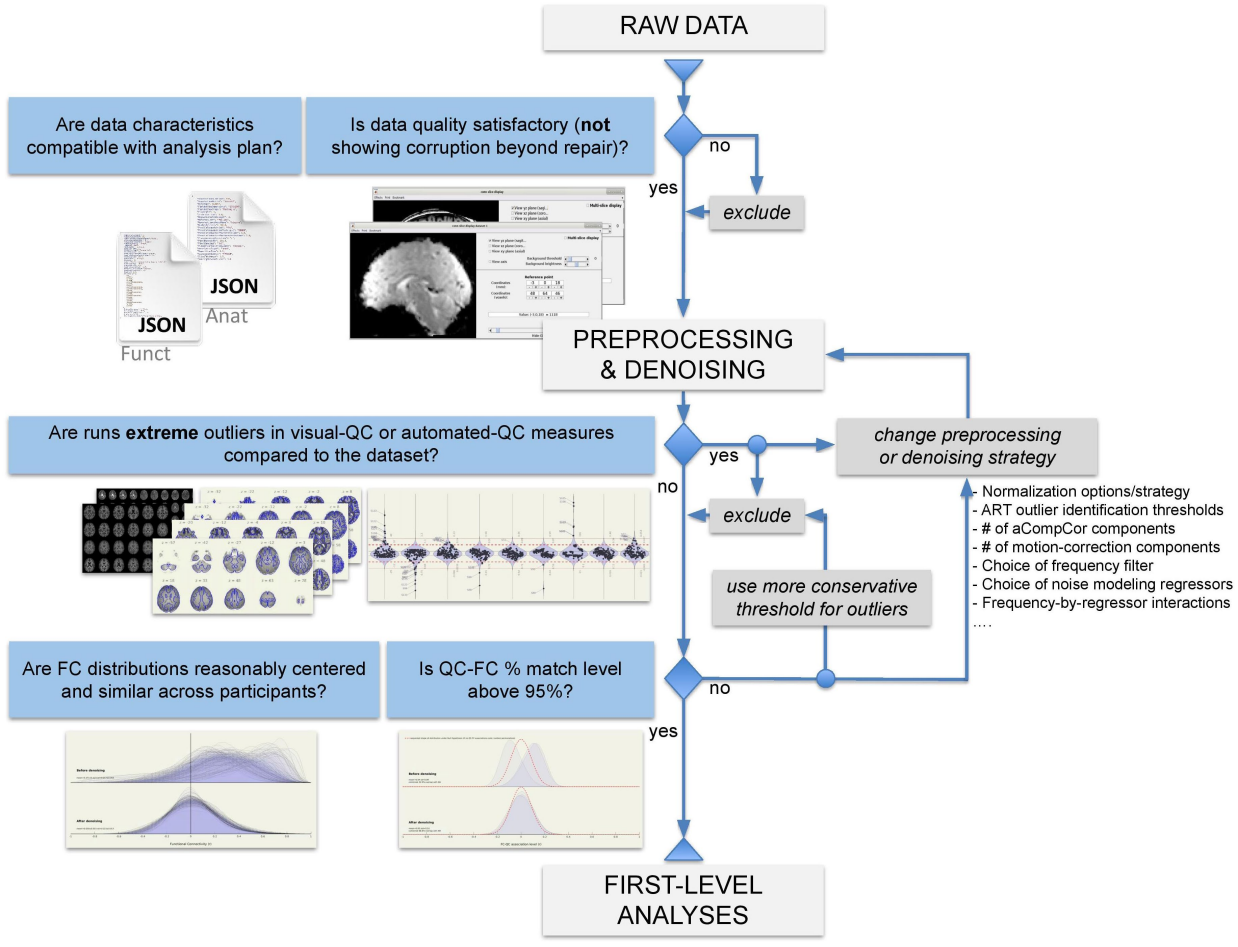
Flowchart of quality control pipeline.

QC of raw-level, preprocessed, and denoised data was carried out following CONN quality control pipeline, building off from Nieto-Castanon (2020, 2022) and summarized in Figure 2.

#### 3.3.1. Quality control of raw-level data

Raw-level functional runs (all slices and all scans) and anatomical images (all slices) were visually inspected using multislice interactive displays of each participant’s data, as well as a combined montage of a single slice across all participants. We also inspected information from json sidecar files and header of nifti files to gather information about image resolution and scanner acquisition parameters. The goal of this step was to familiarize ourselves with the data, identify potential sources of heterogeneity, possible incongruencies among different sites or subjects, and inspect the data for potential outliers or artifacts that may require additional consideration during preprocessing.

#### 3.3.2. Quality control of preprocessed data

Plots of representative brain slices and automated QC measures were generated for each individual subject and functional run to visualize the outputs of preprocessing, identify potential failures of functional and anatomical preprocessing steps, or otherwise confirm that between-run spatial heterogeneity across subjects and runs had been in fact minimized as a result of these steps.

Visual QC included the assessment of the accuracy of functional normalization through the inspection of plots rendering the mean BOLD signal across all scans of the normalized functional data for each participant overlaid onto the 25% boundaries of the gray matter *a priori* probability maps from SPM’s IXI-549 MNI-space template. Similarly, the accuracy of structural normalization was assessed through the inspection of plots displaying each participant’s normalized anatomical images overlaid onto the same gray matter boundaries. Segmentation and anatomical to functional alignment were assessed through plots overlaying the boundaries of each participant’s anatomical GM masks onto the normalized anatomical or functional data.

The presence of potential residual artifacts in functional timeseries was reviewed based on plots displaying a movie of the central axial slice (MNI *z* = 0 mm) of the functional data over time (i.e., over all scans). This movie was rendered above the timeseries traces of (i) the GSC QC timeseries representing scan-to-scan changes in the global BOLD signal, (ii) the FD QC timeseries, characterizing subject motion, and (iii) the outlier QC timeseries, characterizing scans identified as potential outliers. The movies were reviewed to visually assess the amount of motion and imaging artifacts in the data, and identify potential artifacts in the functional data which may not be apparent in the motion, GSC, or outlier timeseries.

Several automated QC summary measures were generated based on preprocessing outputs and related QC timeseries. These measures are described in Table 1 (QC summary measures section). Some of these measures provided an agnostic description of features of the original functional data, including the maximum value of GSC (**MaxGSchange**) and FD (**MaxMotion**). Since, often, these worst-case instances have already been identified as potential outlier scans, these measures inform about the state of the data prior to preprocessing. Other measures such as **MeanGSchange** or **MeanMotion** represent average GSC or FD values limited only to valid (non-outlier) scans, so they can be considered as more informative about the state of the data *after* preprocessing. Other useful statistics include the total number of run-specific outlier scans (**InvalidScans**), the number of non-outlier scans (**ValidScans**), and the proportion of valid scans (**PVS**), providing several indicators of the overall quality and amount of valid data within each individual run for each subject. Last, and aiming to directly quantify the performance of spatial *normalization* and its indirect effect on inter-modality coregistration, the measures **NORM**_func_ (functional normalization) and **NORM**_anat_ (anatomical normalization) measured the similarity between the gray matter mask in the normalized data and in a reference MNI atlas. Relatedly, **AFO** (anatomical to functional overlap) measured the similarity between gray matter masks in functional and anatomical images, evaluating the accuracy of inter-modality coregistration.

Participant-level denoising exclusion criteria included cases that were considered extreme in either the visual QC step, or in the automated QC summary measures. For automated QC summary measures, extreme values were considered those above the threshold Q3 + 3 IQR (or below Q1–3 IQR, for those cases when extreme low values were indicative of problems in the data), where Q1 and Q3 represent, respectively, the first and third quartiles of the distribution of a measure across the entire dataset, and IQR represents their difference (inter-quartile range).

#### 3.3.3. Quality control of denoised data

QC of denoised data aimed at evaluating the quality of the functional data after denoising. Since denoising is the last step when preparing the data before computing functional connectivity measures or performing other statistical analyses, quality control measures of the denoised data provide a way to globally evaluate the suitability of the resulting fMRI data for functional connectivity or other statistical analyses.

Participant-level visual QC aimed at evaluating possible patterns or other features that may be visible in the BOLD signal timeseries after denoising and which may be indicative of a possibly too liberal or too conservative denoising strategy. In particular, we reviewed run-specific plots rendering carpetplots (Power, 2017) of fully preprocessed BOLD timeseries before and after denoising, together with the traces of GSC, FD, and outliers timeseries. These were inspected to confirm that sudden and synchronized variations in signal intensity had been flagged as outliers, and that there are no visible residual large-scale patterns in the BOLD signal timeseries, which could indicate the persistence of global or widespread noise sources (for example, respiratory-related motion or artifacts can appear as patterns with frequency around 0.3 Hz). Carpetplots carry a rich set of information about the timeseries which, in combination with other indicators of potential problems in the data, allow researchers to hypothesize potential sources of noise that may be prevalent in the data, guiding the search of possible solutions.

Several QC summary measures were computed characterizing properties of the BOLD signal after denoising (Figure 1). These measures are described in Table 1 (QC summary measures section). The QC measure **DOF** computes the effective degrees of freedom of the BOLD timeseries after denoising. Lower values (close to zero or negative) indicate that denoising is overly aggressive for the number of functional scans available, and that noise correction comes at the expense of loss of meaningful variability severely impacting our ability to accurately estimate any model parameters of interest from the BOLD timeseries, such as functional connectivity measures or task-related responses. The QC measure **BOLDstd** characterizes the stability of the BOLD signal after denoising. BOLDstd is a measure similar to MeanGSchange but computed from the data after denoising. It is inversely related to the BOLD signal temporal signal-to-noise ratio and, similarly to GCOR, high values are often indicative of the presence of potential noise sources in the residual fMRI data, although it needs to be interpreted with care as unusually low values can also indicate low effective degrees of freedom associated with the loss of meaningful variability from the BOLD timeseries. The QC measure **GCOR** (Global Correlation; Saad et al., 2013) represents the mean of functional connectivity measures (BOLD signal bivariate correlation coefficients) among all voxels, and it has been proposed as an effective control covariate for group-level analyses. GCOR often takes small positive values, caused by local correlations resulting in positive skewness in the distribution of functional connectivity values. High values can indicate an insufficient denoising strategy, and negative values can result from overly aggressive denoising, global signal regression, or biased-inducing denoising strategies.

Additional QC procedures and measures were derived from the distribution of functional connectivity (FC) values, computed as Pearson’s *r* correlation coefficients between the BOLD signal timeseries after denoising among all pairs from a fixed set of 1,000 random voxels within the MNI-space gray matter template mask, in order to evaluate a relatively dense sample of connections from the whole-brain connectome.

Visual inspection of these distributions allowed us to evaluate the relative presence of residual noise sources in the BOLD timeseries of each individual participant, which tend to shift the entire FC distribution toward positive values, altering the FC distribution center (representing the value GCOR) and its overall shape in a manner that is highly variable across different participants and across different runs. In comparison, the relative absence of noise sources is expressed as FC distributions that appear relatively centered (with a small positive distribution mean, and a distribution mode approximately at zero) and similar across different runs and participants.

Participant-level exclusion criteria included severe departures from expected FD distribution shapes after denoising – that is, with significantly skewed, shifted, flat, or bimodal distributions after denoising – as well as the presence of extreme outlier values in any of the computed QC measures (using the same Q3 + 3 IQR or Q1–3 IQR thresholds as before).

Last, the QC measure **QC-FC %** (percent match in QC-FC correlations) represents an individual quality control measure characterizing a property of the entire dataset, rather than properties of individual participants or runs. This measure is also computed from these same distributions of FC values (one FC distribution per participant), but this time focusing on QC-FC inter-subject correlations (Ciric et al., 2017), evaluating whether changes in the spatial correlation structure of the BOLD data covaried with participant-level quality control measures. In particular, using the same sample of connections from the whole-brain connectome estimated in the FC distribution step above, we computed the bivariate Pearson’s *r* correlation coefficients across participants between each of the estimated connectivity values and representative QC measures (MeanMotion, InvalidScans, and PVS). The resulting distributions of QC-FC correlations were evaluated to detect systematic biases by computing the distributional distance between these distributions and those expected by chance (in the absence of QC-FC correlations, as estimated using permutation analyses). QC-FC % values were used to evaluate whether the chosen combination of preprocessing and denoising steps, as well as the choice of thresholds for participant-level exclusion criteria and other QA procedures resulted in satisfactory fMRI data quality levels, and to choose between possible alternatives when necessary. Match levels above 95% were considered indicative of negligible modulations in the BOLD signal correlation structure, while lower values are considered indicative of the persistence of potential problems in the denoised data, requiring either alternative preprocessing and denoising choices or more severe participant exclusion criteria (Figure 2).

## 4. Results

### 4.1. Participants and data characteristics

Information reported here derive from investigating the nifti files characteristics directly or from their sidecar json files, which had been generated prior to release *via* unspecified procedures (*n* = 124) or *via* dcm2niix (Li et al., 2016) v1.0.20170314 (*n* = 15).

In this study, we analyzed resting state and anatomical MRI data from 139 participants acquired from 7 sites, including 151 functional runs and 139 anatomical images (mprage, 3D TFE, or unspecified). All sites contributed 20 participants except for site #3 (*n* = 16) and site #4 (*n* = 23). Throughout the manuscript, individual participants are referred to using both the collection’s ID number (e.g., sub-___) where the first digit reflects the acquisition site of origin, and using ascending numbers (e.g., S___) representing participants ordered from site #1 to site #7.

The fmri-open-qc-rest collection was characterized by data with heterogeneous image resolution, scanner acquisition parameters, and experimental design. A detailed characterization of data features broken down by acquisition site is reported in Supplementary Tables S1, S2 for anatomical images, and in Table 2 for functional data.

**Table 2:** Functional MRI data information for each acquisition site.

	Site #1	Site #2	Site #3	Site #4	Site #5	Site #6	Site #7
*N*	20	20	16	23	20	20	20
Collection ID	Sub-101 to 120	Sub-201 to 220	Sub-301 to 316	Sub-401 to 423	Sub-501 to 520	Sub-601 to 620	Sub-701 to 720
CONN ID	S1 to S20	S21 to S40	S41 to S56	S57 to S79	S80 to S99	S100 to S119	S120 to S139
MRI scanner	Philips Achieva	Philips Achieva	Philips Achieva DS	/	Philips Achieva (5) Siemens Trio Tim (14) Siemens Prisma Fit (1)	Siemens Magnetom Trio	Siemens Verio
Head coil	/	/	32 channels	/	/	12 channels	/
Flip angle [°]	75	90	90	/	90 (17) 80 (3)	90	80
Phase encoding direction	j-	j-	j-	/	j- (15) / (5)	/	j-
Parallel acquisition technique	SENSE	SENSE	SENSE	/	/ (15) no_stimulation SENSE (5)	/	/
Voxel dimension [mm^3^]	2.7×2.7×3 (19) 2.3×2.3×3 (1)	3x3x3.8	1.6×1.6×3.1	2.7×2.7×3	3×3×4 (15) 1.9×1.9×4 (5)	4×4×4	3×3×3.5
Field of view [slices]	96×96×47 (19) 112×112×47 (1)	80×80×38	128×128×45	96×96×47	80×80×35 (10) 128×128×34 (5) 80×80×34 (4) 80×80×39 (1)	64×64×32	64×64×39
Repetition time [s]	2.5	2	2.5	2.5	2	2.5	2.5
Acquired EPI runs	1	1	1	1	1	1 (8) 2 (12)	1
Scans acquired	156 (18) 128 (2)	150	162	123	144	[240–724]	198
Acquisition duration [s]	390 (18) 300 (2)	300	405	307.5	288	[600–1,810]	495
Slice timings available	Yes	Yes	Yes	/	Yes (13) / (5) wrong (2)	/	Yes
Task stimuli	White cross over black screen	Eyes closed	White cross over black screen	/	Eyes open	/	Eyes closed
Task instructions	/	Rest	Relax and think of nothing particular	/	/	/	/
Number of properties present in json file(s)	31	29	32	2	15 (5) 20 (13) 21 (2)	8 (8) 8 each run (12)	14

The information reported refers to all participants of each site, unless otherwise specified by the number in parentesis reflecting the subset of participants. Participants are identified by the collection’s ID number (e.g., sub-___) and by increasing numbers (e.g., S___) representing participants in ascending order. mm, millimeters; MRI, magnetic resonance imaging; properties of a json file, key-value pairs included in the json files; s, seconds; SENSE, sensitivity encoding; °, degrees; “/” indicates that information was not available.

Gathered information about functional data suggested that data were acquired by Siemens or Philips MRI scanners of various models (Trio Tim, Prisma Fit, Verio and Magnetom Trio, or Achieva or Achieva DS), using head coils with 12, 32, or unspecified number of channels. Data sampling differed on temporal (2- or 2.5-s TR) and spatial parameters, such as voxel dimensions (ranging from 1.6 × 1.6 × 3.1 to 4 mm isotropic) and number of acquired slices (between 32 and 45). No information was available regarding whether any online processing was performed during or after acquisition, for example prospective motion correction or denoising. By design, the experience of the participants was also different. Total time spent for the functional BOLD imaging acquisition ranged between 288 and 1,810 s (approximately between 5 and 30 min) which was acquired either in one continuous run or split into two (*n* = 12). During the functional data acquisition, participants were exposed to different visual stimuli (black screen with crosshair, eyes closed, or unspecified) and instructions (rest, relax and think of nothing particular, or unspecified).

Information incongruencies were encountered for sub-506 (S85) and sub-507 (S86) functional data, wherein 39 slice timings were reported in the sidecar json files but only 35 slices were available as per the nifti header information. This may suggest that these functional runs were not in a raw-level form or that the json files included faulty information.

There was no available information regarding several elements which had been shown to carry meaningful individual differences and which were relevant for data interpretation. No information was available regarding participant demographics (age, sex, medical and mental health history, mental and physical status at time of acquisition, psychoactive medication, etc.), participant inclusion and exclusion criteria, informed consent and assent. For example, the task description of sidecar json files of site #1 could be interpreted as suggesting that participants might include children who were asked to withhold taking psychostimulants the day prior to and the day of scanning; and the procedure description reported from the json files of site #5 could imply that participants were recruited under a study of brain traumas. Additionally, no information was available about the study paradigm, study design, or presence of experimental manipulation prior to or during data acquisition. Relatedly, it was not possible to determine whether the same individual was scanned in different sites or longitudinally, or if data were deemed unusable by the experimenters for any reason.

Critically, we did not know whether all or any of the above elements covaried with site and, consequently, whether potential inter-site variability encompassed meaningful individual differences in addition to heterogeneity associated with differences in scanner or acquisition details. Given the information available, or lack thereof, *site* was identified as a control variable. We cannot rule out that differences among sites may include meaningful factors, such as sample’s age, health or medical status, or study design. These may legitimately affect BOLD signal properties of interest, including functional connectivity measures, in a manner that cannot be effectively separated from other sources of differences among sites, such as those resulting from differences between MR acquisition parameters or noise sources. Because of this, whenever possible we limited analyses of intersubject variability to focus only on within-site analyses, explicitly disregarding variability across sites due to the unavoidable issues when attempting to interpret sources of inter-site variability.

### 4.2. Raw-level data QC

**Figure 3:**
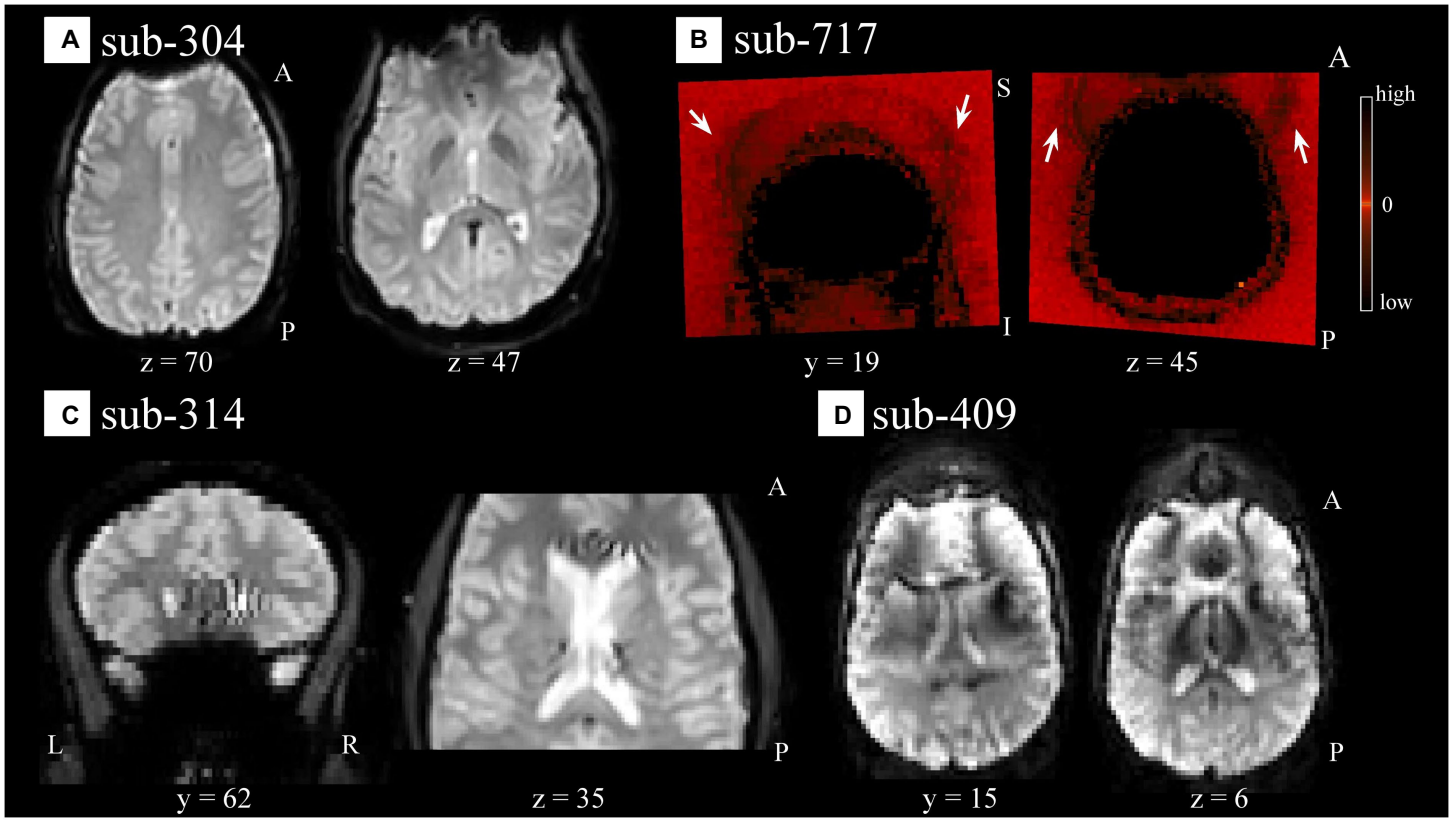
Spatial artifacts of raw-level functional data. **(A)** Spatial distortions and signal drop out in superior/orbito-frontal regions in sub-304 (S44). **(B)** Aliasing or ghosting in the coronal (*y* = 19) and axial (*z* = 45) slices from sub-717 (S136). For visualization purposes only, intensity values have been scaled so that low and high values would appear darker, making more evident artifacts such as those highlighted by the white arrows stemming from the superior (left image) and frontal (right image) regions of the head. **(C)** Unspecified signal inhomogeneity artifacts affecting sub-314 (S54) functional scans localized near areas of high intensity contrast such as CSF to WM. **(D)** Ghosting, spatial distortions, and signal inhomogeneities are noticeable in sub-409 (S65) functional data across all scans and several slices. For all panels, the images render the first functional scan of raw-level data.

Visual QC of the functional data identified different types of artifacts. We noticed artifacts appearing as spatial susceptibility distortion or signal drop out (e.g., sub-304 [S44]; Figure 3A), ghosting/aliasing (e.g., sub-717 [S136]; Figure 3B), signal inhomogeneity localized in regions of high tissue contrast [e.g., sub-314 (S54); Figure 3C], of unspecified nature, or their combination [e.g., sub-409 (S65); Figure 3D]. For a complete list of identified artifacts broken down by participant and modality see Supplementary Table S3.

Incorrect orientation of functional data was encountered for sub-518 (S97) and sub-519 (S98), which appeared upside-down. We considered to correct it by either applying a 180° rotation along the y-axis (i.e., preserving the relative position between the x, y, z axes) or a non-rigid reflection along the z-axis (i.e., flipping the data *via* a x, y, −z axis transformation which effectively would swap the signal between the left and right hemispheres). We opted to flip the data in both instances, based on the better visual match achieved between the flipped functional data and its respective anatomical data (Supplementary Figure S1).

**Figure 4:**
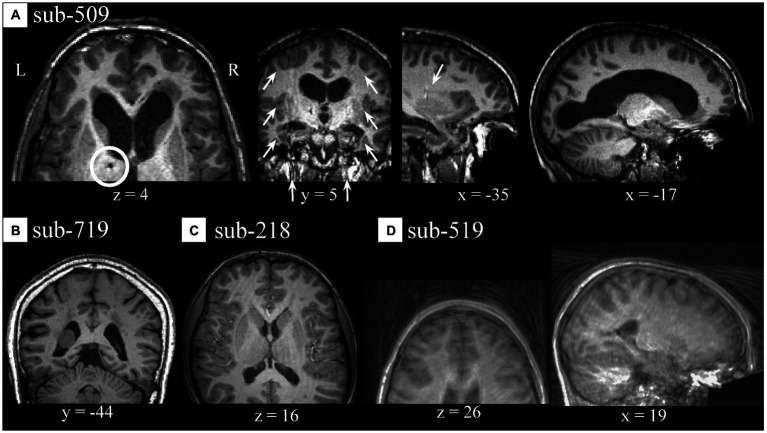
Spatial artifacts of anatomical raw-level data. **(A)** Sub-509 (S88) presented signs of potential past surgery (*z* = 4) appearing as dark, small, localized areas traveling through several slices, signal intensity inhomogeneity localized bilaterally along the *y*-axis (*y* = 5 and *x* = −35), and individual anatomical variations of size and shape of the lateral ventricles (*x* = −17). **(B)** Individual anatomical differences in the form of an asymmetrical mass or unspecified signal inhomogeneity localized in the lateral ventricle of a sub-719 (S138). **(C)** Motion-related artifacts or ringings in sub-218 (S38). **(D)** Sub-519 (S98) showed severe aliasing, ghosting, and/or motion-related artifacts.

During visual QC of anatomical data, we noticed few artifacts. Several participants from site #5 showed potential signs of past surgeries, as identified by localized darker areas (appearing as dots) traveling through contiguous slices reaching from the cortex to subcortical medial areas [e.g., sub-509 (S88); Figure 4A, *z* = 4]. Often, these artifacts were localized in areas which appear to correspond to artifacts in the participant’s functional data (Supplementary Figure S2). Sub-509 (S88) showed areas of intensity inhomogeneities bilaterally (Figure 4A, *y* = 5 and x = −35) which appeared as bands in the y axis, and large asymmetrical lateral ventricles (Figure 4A, *x* = −17). Other cases of potential anatomical variations or artifactual signal intensity were encountered including in sub-719 (S138; Figure 4B). Few cases of ringing-like patterns more prominently visible along the z-axis were noticed in a sub-218 (S38; Figure 4C) and in a few other anatomical images (see Supplementary Table S3). Additionally, there were few cases with noticeable motion-related and ghosting, of which sub-519 (S98; Figure 4D) was an example. Inasmuch the preprocessing of anatomical images for FC-MRI analysis was instrumental to preparing the functional data, a low(er) quality of anatomical images was not considered a major roadblock unless it produced a faulty segmentation or normalization.

During anatomical visual QC, we also observed what could be described as a skin marker on the forehead (right hemisphere) of most participants from site #5 (*n* = 15) including all those scanned with Philips Achieva, and in a few from site #7. While there was no available information regarding which hemisphere the marker was placed on, and under the assumption that they would be placed in a standardized fashion, the consistent lateralization with which the marker was observed for all participants was considered as a hint of lack of left–right flip relative to one another.

**Figure 5:**
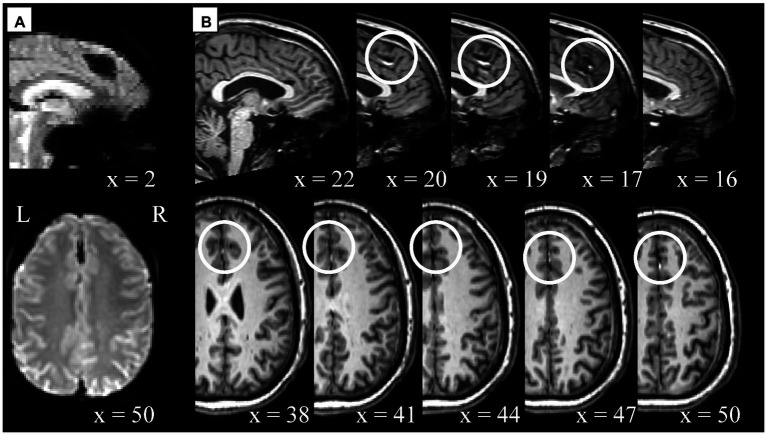
Example of cross-modality visual quality control for artifact characterization. Potential artifact of unspecified type in sub-315 (S55) functional (panel **A**) and anatomical (panel **B**) data. **(A)** Signal inhomogeneity affecting several axial slices localized in the interhemispheric fissure. The first scan is displayed here, however similar artifacts are noticeable across all scans. **(B)** Unspecified anatomical artifacts rendered in contiguous sagittal (*x* = 22 to *x* = 16) and axial slices (*x* = 38 to *x* = 50) in the top and bottom row, respectively. White circles indicate areas where artifacts are visible in a location comparable between functional and anatomical data. Note, the anatomical and functional images displayed here were in raw-level form, hence the spatial coordinates refer to subject-space and might differ across modalities.

Cross-modality visual comparison aided the characterization of artifacts. For example, unspecified signal intensity inhomogeneity was noticed in the functional data of sub-315 (S55; Figure 5A, *x* = 2), which corresponded to an undefined artifact or anatomical feature (Figure 5B). The artifact was localized in the medial-superior area above the cingulate cortex in the interhemispheric fissure, appearing dark in the functional data and bright in the anatomical images. Additionally, several examples of highly localized signal inhomogeneity with sharp intensity differences were characteristic of participants from site #5. From a visual inspection, those appeared similar to those reported in Figure 5, but the comparison with the anatomical data suggested that those could potentially derive from past brain surgeries (e.g., sub-509 [S88]; Supplementary Figure S2).

Overall, only one run corresponding to sub-409 (S65) was deemed to be excluded based on extreme spatial corruption severely affecting multiple slices and persistent across all scans. All other cases mentioned above were flagged as uncertain (see Supplementary Table S3 for a complete list) as we considered that in the absence of additional indications their potential effect on the quality of the BOLD signal may not be severe enough to warrant exclusion.

### 4.3. Preprocessed data QC

Since fieldmaps were not available, our preprocessing included a direct, rather than indirect, normalization procedure to try to minimize EPI-specific warping caused by susceptibility distortions (Calhoun et al., 2017). Similarly, we skipped STC because slice timing information was available for only a portion of runs (*n* = 89 out of 151) and most importantly, it was selectively missing for entire sites (#4, #6, and some cases from site #5). We elected to skip STC for all participants in order to prevent introducing variability driven by distinct analytical approaches into the results, which, in light of the characteristics of the fmri-open-qc-rest collection, could exacerbate potential inter-site (and in the case of site #5, even intra-site) heterogeneity even further.

**Figure 6:**
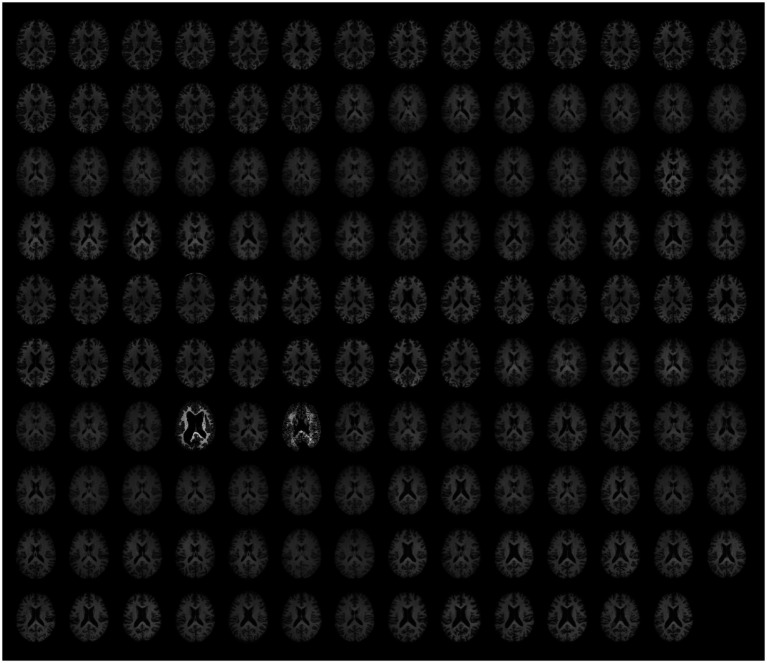
Preprocessed anatomical data. The same axial slice (MNI *z* = 18) of the fully preprocessed anatomical images is rendered for each participant (*n* = 139). For visualization purposes only, the BOLD signal intensity was scaled by the average value within each image.

**Figure 7:**
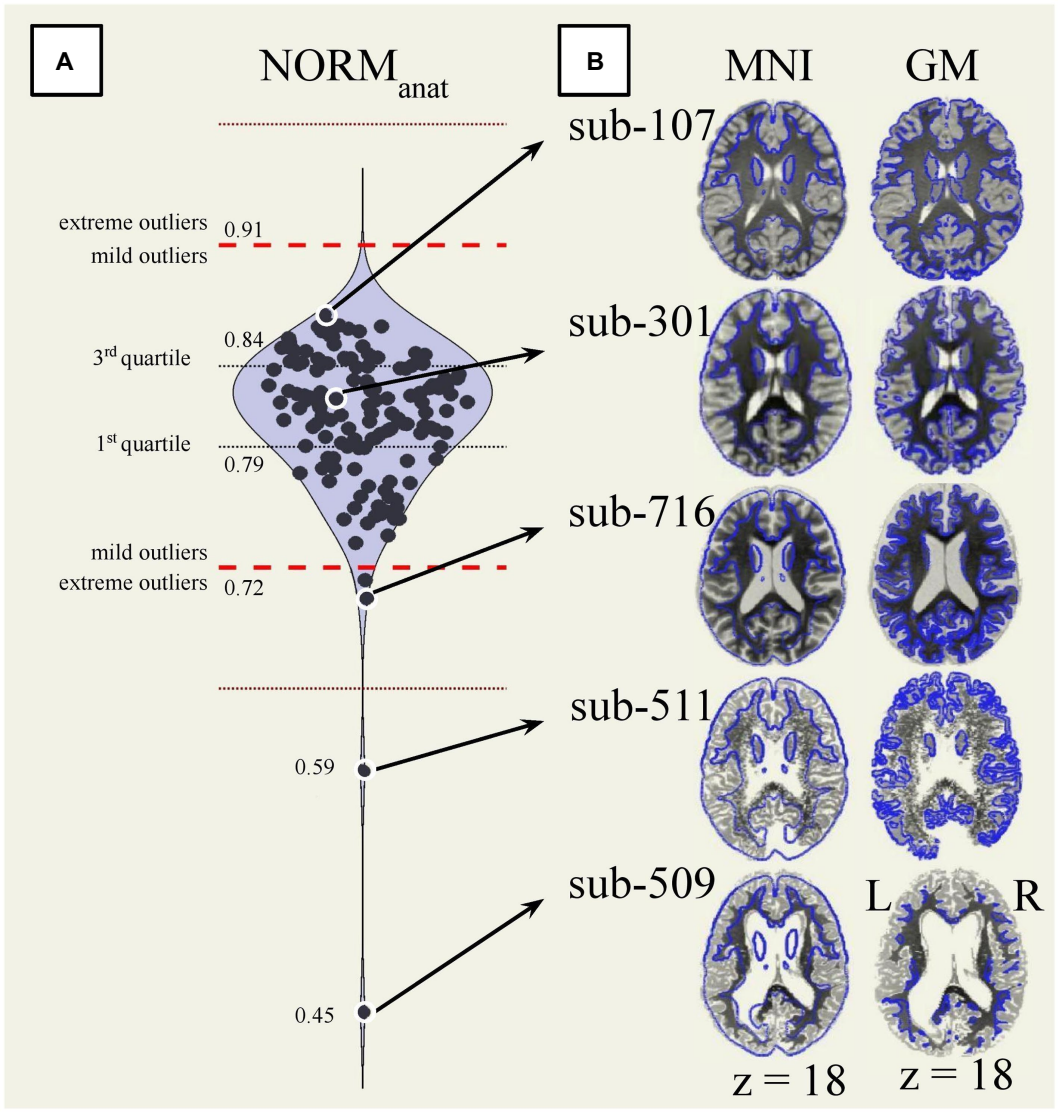
Automated and visual quality control of normalized anatomical data. **(A)** Distribution of the overlap between the normalized anatomical data (*n* = 139) and the MNI-space (NORM_anat_). Extreme outliers are identified as values 3 IQR above the 3rd quartile or below the 1st quartile (red dotted lines). Mild outliers are values 1.5 IQR above the 3rd quartile or below the 1st quartile (red dashed lines). **(B)** The same reference axial slice (MNI *z* = 18) renders the normalized anatomical images from five participants. The participants’ anatomical image is, on the left, overlaid on the 25% boundaries of the gray matter *a priori* probability maps MNI-space template (blue outline), and on the right, against each participant’s anatomical gray matter boundaries. The participants reported in the figure are ordered from top to bottom based on their NORM_anat_ values. Specifically, compared to the full dataset, sub-107 (S7) had the highest value, sub-301 (S41) was close to the median value, sub-716 (S135) was close to the low mild outlier threshold, sub-511 (S90) and sub-509 (S88) were the two lowest values and extreme outliers. GM, gray matter; MNI, Montreal Neurological Institute space; NORM_anat_, overlap between the MNI-space and the normalized anatomical data.

Visual QC of preprocessed data identified severe failures of anatomical normalization and segmentation for sub-509 (S88) and sub-511 (S90). In both cases, the normalized anatomical and segmented tissue ROIs appeared fragmented and showed poor continuity within tissue type but sharp differences across tissues, see Figure 6 (slices in row 7 columns 4 and 6) and Figure 7B (bottom).

**Figure 8:**
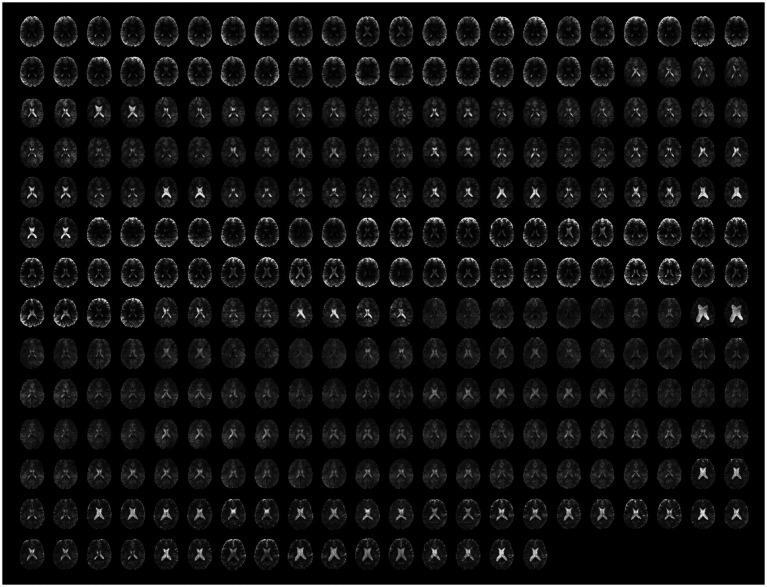
Preprocessed unsmoothed functional data. The same axial slice (MNI *z* = 18) for the first and the last functional scan are rendered for all runs (*n* = 151). For visualization purposes only, the BOLD signal intensity of each scan was scaled by its average value.

Beyond those issues, visual inspection of the functional and anatomical data and potential residual artifacts in the functional timeseries identified no other obvious failures of functional preprocessing, including for the cases flagged as uncertain during raw-level data QC. For an overview of the full dataset after preprocessing, see Figure 6 (anatomical images, *n* = 139) and Figure 8 (functional scans, *n* = 151).

**Figure 9:**
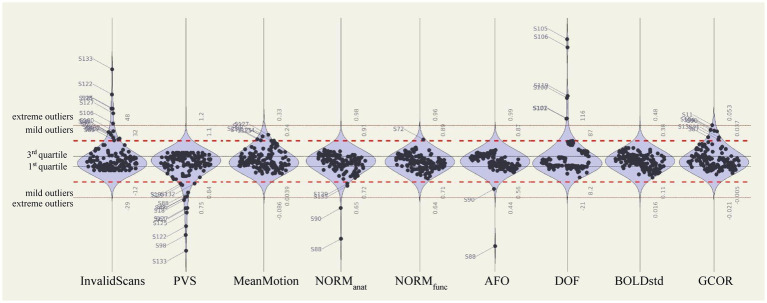
Automated quality control measures of preprocessed and denoised data. Distributions of automated QC measures extracted from the full fmri-open-qc-rest collection (*n* = 139 anatomical and *n* = 151 functional data). QC measures were calculated from preprocessed functional (InvalidScans, PVS, MeanMotion, NORM_func_, and AFO), preprocessed anatomical (NORM_anat_ and AFO), and denoised functional (DOF, BOLDstd, and GCOR) data. Extreme outliers were identified as values 3 IQR below the 1st quartile or above the 3rd quartile (red dotted lines). Mild outliers were defined as values 1.5 IQR below the 1st quartile or above the 3rd quartile (red dashed lines). BOLDstd, standard deviation of the BOLD signal; DOF, degrees of freedom; GCOR, global correlation; IQR, interquartile range; NORM_func_, MNI-space template to functional overlap; NORM_anat_, MNI-space template to anatomical overlap; PVS, proportion of valid scans.

Automated QC measures (InvalidScans, PVS, MeanMotion, NORM_anat_, NORM_func_, and AFO in Figure 9; other measures are reported in Supplementary Figure S3) were generated from *n* = 151 functional runs and *n* = 139 anatomical images (Figure 9, left). Low extreme outliers (values 3 IQR below the 1st quartile) were identified for NORM_anat_ [*n* = 2, sub-509 (S88) and sub-511 (S90)] and AFO [*n* = 1, sub-509 (S88)], which corresponded to the cases identified during visual inspection. These data were also identified as extreme low outliers based on the distribution of total tissue volumes (Supplementary Figure S4). We visually inspected again the cases identified as mild low outliers from the distribution of NORM_anat_ (*n* = 2; see sub-716 [S135] in Figures 7A,B), NORM_func_ (*n* = 0), and AFO (*n* = 0) and confirmed that those indicated an acceptable preprocessing performance.

Several extreme low PVS outliers were identified (*n* = 7 with PVS below 75%): sub-118 (S18), sub-405 (S61), sub-519 (S98), sub-703 (S122), sub-706 (S125), sub-708 (S127) and sub-714 (S133) as well as several, mostly overlapping, extreme high InvalidScans participants (*n* = 6 with 48 or more InvalidScans): sub-519 (S98), sub-607 (S106), sub-703 (S122), sub-706 (S125), sub-708 (S127) and sub-714 (S133). The only participant with extreme high InvalidScans who did not have low PVS was sub-607 (S106), who, despite having 50 outlier scans, accounted for less than 7% of the total scanning session.

One participant [sub-111 (S11)] had a GCOR value (0.0534) borderline but below the level of extreme outlier (GCOR = 0.0535). However, this participant showed no obvious artifactual effects in carpetplots, or from other visual checks, nor had values in the mild (1.5 IQR) or extreme (3 IQR) outlier range for any other QC measure. Given that GCOR potentially includes some amount of meaningful intersubject variability, we elected not to exclude this run in order to avoid suppressing possibly natural variability.

Last, confirming our previous observations, there were strongly significant differences in all QC measures between the different sites (InvalidScans *F*(6,132) = 4.24 *p* = 0.0006, PVS *F* = 3.33 *p* = 0.0044, MeanMotion *F* = 8.85 *p* < 0.0001, NORM_anat_ *F* = 13.22 *p* < 0.0001, NORM_func_ *F* = 23.49 *p* < 0.0001, and AFO *F* = 13.42 *p* < 0.0001).

### 4.4. Denoised data QC

The distribution of automated QC measures (DOF, BOLDstd, and GCOR) for all denoised data (*n* = 151 corresponding to 139 participants) is reported in Figure 9 (right). There were no extreme outliers in BOLDstd, nor extreme low absolute DOF values, and participants with the lowest DOF values in this dataset [sub-519 (S98) DOF = 17.1, sub-405 [S61] DOF = 24.2, and sub-714 (S133) DOF = 26.2] were already identified as extreme outliers with low PVS values. As with preprocessing QC measures, there were strongly significant differences in all QC denoising measures evaluated when compared between the different sites [DOF *F*(6,132) = 27.92 *p* < 0.0001, BOLDstd *F* = 19.65 *p* < 0.0001, and GCOR *F* = 12.98 *p* < 0.0001].

**Figure 10:**
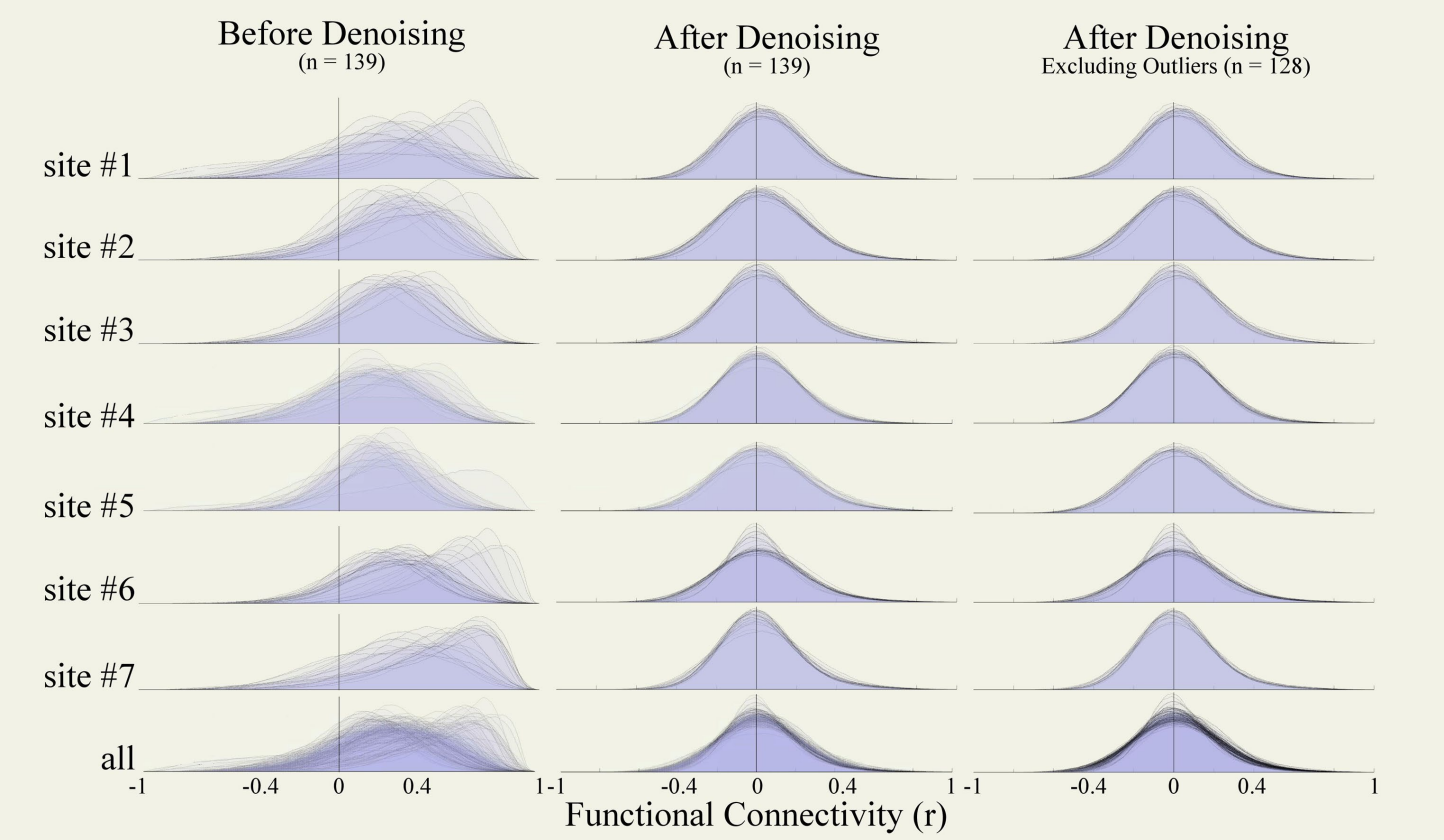
Functional connectivity density distributions. Density distributions of within-run FC strengths (*r* coefficients) between all pairs among 1,000 randomly selected voxels from functional runs of the entire data collection (*n* = 139) before (left) and after denoising (central), and after excluding outlier runs (right, *n* = 128). FC distributions are plotted for data from each site independently (top) and from all sites jointly (bottom row). FC, functional connectivity.

After preprocessing but before denoising, the distributions of functional connectivity estimates (FC distributions, Figure 10 left column) revealed severe biases, with connectivity values centered at *r* = 0.27 on average across all participants, and also showed high levels of variability in the FC distribution center, with standard deviation 0.12 across participants. After denoising, the FC distributions (Figure 10, central column) were centered around *r* = 0.031, and had low variability (standard deviation 0.01 across participants). Visually, FC distributions after denoising appeared more centered and similar across participants, and nearly symmetrical with slightly longer positive than negative tails, as expected (for comparison, Supplementary Figure S5 displays examples of FC distributions that could result if our denoising strategy had been overly or insufficiently aggressive in this same dataset).

No individual runs were identified as potential outliers after denoising from visual inspection of these results. Site #6 included several runs with distinctive narrower FC distributions, but these were associated with scanning length that were considerably longer (identified in the Figure 9 DOF distribution as having significantly higher degrees of freedom compared to other runs in this dataset). We did not exclude these runs but depending on the planned analyses it may be advisable to consider homogenizing the scanning duration length of the fMRI data.

**Figure 11:**
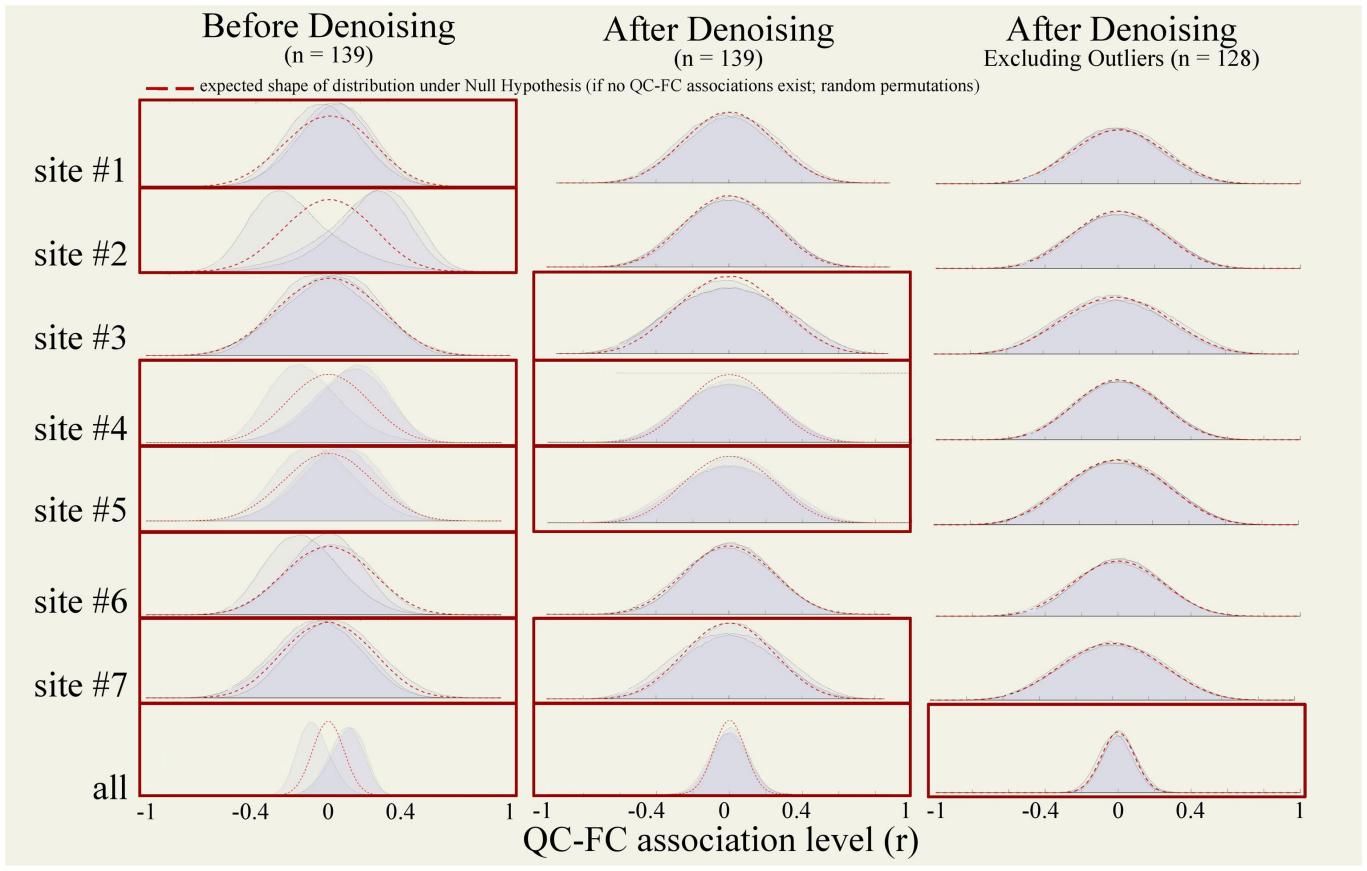
QC-FC correlation distributions. QC-FC plots tested functional connectivity associations with three nuisance factors (MeanMotion, InvalidScans, and PVS). Plots were generated from functional data from all participants (*n* = 139) before (left) and after (middle) denoising, and after excluding outlier runs (right) identified during raw-level, preprocessed, and denoised data QC (*n* = 128). Analyses were performed within each site independently (top) and across all sites jointly (bottom row). Red boxes indicate QC-FC with at least one QC-FC distribution that did not reach above the 95% cutoff. Red dotted lines represent a theoretical artifact-free null-hypothesis distribution. QC, quality control; FC, functional connectivity.

QC-FC correlations were estimated separately within each site to avoid potential site confounder effects. Before denoising, QC-FC correlation distributions showed poor percentage match levels, indicating the persistence of motion and data quality effects on functional connectivity estimates after preprocessing. Specifically, percentage match levels were below the 95% cutoff for InvalidScans [average within-site %match = 86.70 ± 11.77 ranged (65.82; 97.59)], MeanMotion [85.37 ± 13.94 (56.78; 98.52)], and PVS [83.70 ± 11.51 (65.82, 97.59)], see Figure 11 (left) and Table 3 (top).

**Table 3:** FC density distributions and QC-FC correlations.

Site	*n*	*n* excluded	FC mean ± SD	InvalidScans-FC	MeanMotion-FC	PVS-FC	QC-FC performance
Before denoising (*n* = 139)							
Site #1	20	/	0.27 ± 0.13	90.81	91.38	92.01	Below cutoff
Site #2	20	/	0.29 ± 0.08	65.82	56.78	65.82	Below cutoff
Site #3	16	/	0.24 ± 0.07	**97.59**	**98.52**	**97.59**	
Site #4	23	/	0.17 ± 0.08	75.85	78.41	75.85	Below cutoff
Site #5	20	/	0.2 ± 0.07	87.58	91.03	87.58	Below cutoff
Site #6	20	/	0.32 ± 0.13	**97.46**	91.71	75.24	Below cutoff
Site #7	20	/	0.39 ± 0.11	91.78	89.76	91.78	Below cutoff
All	139	/	0.27 ± 0.12	55.18	58.83	64.64	Below cutoff
After denoising (*n* = 139)							
Site #1	20	/	0.04 ± 0.02	**95.73**	**98.89**	**95.96**	
Site #2	20	/	0.04 ± 0.01	**97.19**	**97.02**	**97.19**	
Site #3	16	/	0.03 ± 0.01	92.35	**96.12**	92.35	Below cutoff
Site #4	23	/	0.02 ± 0.01	93.01	**96.07**	93.01	Below cutoff
Site #5	20	/	0.03 ± 0.02	92.27	**95.64**	92.27	Below cutoff
Site #6	20	/	0.03 ± 0.01	**97.68**	**97.04**	**97.26**	
Site #7	20	/	0.02 ± 0.01	91.47	**96.99**	91.47	Below cutoff
all	139	/	0.03 ± 0.01	91.41	94.20	90.02	Below cutoff
After denoising and excluding outliers (*n* = 128)							
Site #1	19	1	0.04 ± 0.02	**96.40**	**97.60**	**96.48**	
Site #2	20	0	0.04 ± 0.01	**97.19**	**97.02**	**97.19**	
Site #3	15	1	0.03 ± 0.01	**95.92**	**97.00**	**95.92**	
Site #4	21	2	0.02 ± 0.01	**98.00**	**98.01**	**98.00**	
Site #5	17	3	0.03 ± 0.02	**98.48**	**98.50**	**98.48**	
Site #6	20	0	0.03 ± 0.01	**97.68**	**97.04**	**97.26**	
Site #7	16	4	0.02 ± 0.01	**97.47**	**99.21**	**97.47**	
All	128	11	0.03 ± 0.01	**97.24**	**95.84**	93.97	Below cutoff

Values reported under FC mean represent the average ± standard deviation across participants of GSC, the mean values of the FC density distributions, and QC-FC represent the percentage match level values, characterizing the presence of inter-subject associations between functional connectivity and subject motion or outlier prevalence. Bold font indicates % match values that are above the 95% cutoff. QC-FC performance values indicate whether any QC-FC measure percentage match level is below the 95% cutoff. FC, functional connectivity; GSC, global signal change; PVS, proportion of valid scans; QC, quality control.

Denoising increased the percentage match levels of QC-FC distributions (Figure 11 middle and Table 3 middle) for InvalidScans [average within-site % match = 94.24 ± 2.56 (91.47; 97.68)], MeanMotion [96.82 ± 1.07 (95.64; 98.89)], and PVS [94.21 ± 2.50 (91.47; 97.26)]. Despite this, several QC-FC correlations still did not pass the desired 95% cutoff for at least one of the three evaluated QC measures, including site #3, site #4, site #5, and site #7 (Table 3).

Excluding all runs with identified extreme outliers in any of the evaluated QC measures (*n* = 10, 1 run identified during raw-level visual QC, 2 runs with problems in spatial normalization, and 7 runs with extreme low PVS) increased the percentage match level of QC-FC distributions for InvalidScans [average within-site % match = 96.79 ± 2.07 (92.35; 98.48)], MeanMotion [97.64 ± 1.03 (96.12; 99.21)], and PVS [96.75 ± 2.04 (92.35; 98.48)]. Despite this, QC-FC correlations of site #3 still did not pass the desired 95% cutoff. Since the distribution of PVS did not show a clear cutoff among those participants with extreme outliers and those with mild outliers, we decided to re-evaluate QC-FC correlations varying the PVS threshold used for participant-level exclusion, excluding one additional participant at a time, in order to identify the minimal number of excluded participants that would result in suprathreshold QC-FC match levels for all QC measures. The results indicate that removing one additional participant, i.e., the 8 participants with lowest PVS values (instead of 7 when using the originally suggested extreme-outliers threshold), was sufficient to push all sites above the desired 95% threshold in QC-FC match levels across InvalidScans [97.3 ± 0.89 (95.92; 98.48)], MeanMotion [97.77 ± 0.86 (97; 99.21)], and PVS [97.26 ± 0.86 (95.92; 98.48)], see Figure 11 (right column) and Table 3 (bottom). Automated QC measures of the final *n* = 11 excluded participants and their carpetplots are reported in Supplementary Figures S6 and S7, respectively.

## 5. Discussion

In this study, we presented the CONN quality control pipeline (Table 4; Figure 2) based on a combination of visual and automated QC procedures. Publicly available resting state data were analyzed to showcase a complete QC workflow for the screening of raw-level, preprocessed, and denoised data for volume-based FC-MRI analysis. This pipeline includes visual-QC steps, where researchers visually judge the severity of potential artifacts in the raw, preprocessed, and denoised data, as well as a number of automated QC measures quantifying relevant aspects of the functional data. We recommend that researchers use the combination of visual- and automated- QC measures to motivate possible changes in their data preprocessing or denoising strategy that would address the issues raised by these measures, or, ultimately, to determine a list of individual participants or runs that may need to be excluded from the main analyses. The choice of a threshold for participant exclusion should be informed by the characteristics of one’s own sample and the needs of their research questions or planned analyses. Rather than using absolute thresholds in QC measures, we suggest that sample-specific thresholds, such as the choice of a classical “extreme outliers” threshold of Q3 + 3 IQR for extreme high values (or Q1–3 IQR for extreme low values) are a reasonable starting point for participant exclusion. Last, our QC workflow uses the measure QC-FC %, characterizing the presence of inter-subject associations between functional connectivity and subject motion or outlier prevalence, and the stability of the FC distributions across different runs and participants (FC mean ± SD), as a way to evaluate the overall quality of the data, helping guide possible choices between alternative preprocessing and denoising strategies or participant exclusion thresholds.

**Table 4:** CONN quality control pipeline checklist and exclusion criteria for whole brain resting state functional connectivity analysis.

	Category	QC Checklist	Tools	Exclusion criteria
Raw-level data	Source of heterogeneity of no interest (defined by the data intended used)	Acquisition parameters	MRI data	(A) Data that do not meet criteria for the specific analysis goals as defined by each individual research study
		Demographic	Sidecar json files	
		Task design	Scan sequences protocol	
	Artifacts	Ghosting	Visual inspection (scan-to-scan and slice-to-slice)	(B) Data corrupted beyond repair as judged by rater
		Aliasing		
		Foreign objects artifacts		
		Dropouts/truncation		
		Ringing		
		Spatial distortions		
		Contrast inhomogeneities		
	Personalized preprocessing needed	Artifacts that may require personalized consideration	Visual inspection (slice-to-slice)	
	Challenging data features	Motion related artifacts Anatomical variations	Visual inspection (scan-to-scan and slice-to-slice)	
Preprocessing	Failures of functional preprocessing	Artifacts in the timeseries	Visual comparison between the scan-to-scan movie of a reference functional slice with motion, GSC, and outlier timeseries traces	
		Normalization	Visual comparison between normalized functional data and MNI template	(C) † Functional data which cannot be preprocessed satisfactorily as judged by rater
			Visual comparison between anatomical gray matter and normalized functional data	
			Automated QC measure NORM_func_	(D) † Cases with extreme values, as judged by a sample-specific Q1-3 IQR threshold criterion
	Failures of anatomical preprocessing	Normalization and segmentation	Visual comparison between normalized anatomical data and MNI template	(E) † Anatomical data which cannot be preprocessed satisfactorily as judged by rater
			Visual comparison between anatomical gray matter and normalized anatomical data	
			Automated QC measures AFO and NORM_anat_	(F) † Cases with extreme values, as judged by a sample-specific Q1-3 IQR threshold criterion
Denoising	Residual noise factors	Within-participant	Visual comparison of carpetplots with motion, GSC, and outlier timeseries traces	
		Between-participant	Other QC variables: distribution of participant-level QC measures	(G) † Cases with extreme values in PVS, MeanMotion, or DOF, as judged by a sample-specific Q3 + 3 IQR or Q1-3 IQR threshold criterion
			Distribution of functional connectivity values	(H) † Extremely skewed, shifted, flat, or bimodal functional connectivity distributions after denoising, as judged by rater.
				Also used to guide preprocessing, denoising, and participant-exclusion-criteria choices.
			Distribution of QC-FC associations, for InvalidScans, MeanMotion, and PVS	Used to guide preprocessing, denoising, and participant-exclusion-criteria choices.

Cases with extreme values could be represented by values below 3 times the interquartile range above the 3rd quartile or below the 1st quartile, depending on the specific QC measure, compared to the full dataset distribution. BOLD, blood oxygenation level-dependent; FC, functional connectivity; GCOR, global correlation; MNI, Montreal Neurological Institute; QC, quality control; TPM, tissue probability map. † Indicates exclusion criteria applied only if potential remediatory analytical or processing alternatives fail.

Our QC workflow included a combination of procedures, of which some can be quantified precisely and even automated, while others cannot and will ultimately rely on each researcher’s experience and judgment. In both cases, our approach is not that there is an “optimal” or even “correct” form of QC, but rather to encourage researchers to understand the rationale behind performing QC, follow a reasonable set of procedures, justify their choices during QC, and report their decision process when sharing their results to the community. For example, there is currently no agreed-upon correct choice or criterium of what constitute severe ghosting or other image artifacts, but our recommendation is for researchers to perform visual QC to evaluate the presence and severity of artifacts in their data, and then to define, based on their own criteria, experience, research goals, and specificities of their sample, what constitutes possibly extreme cases that would justify their exclusion. From this general perspective, we have attempted to provide specific measures and thresholds that could be used as precise exclusion criteria when possible (as sample-specific outliers, using a Q3 + 3 IQR threshold for individual QC measures, and as an absolute 95% threshold in QC-FC percent match levels), while also leaving room for other less easily quantifiable aspects of QC (using severity scores based on a researcher’s own criteria during visual QC, and judging the overall level of centering and similarity of the QC distributions across the different subjects in our sample).

In that context, several automated QC measures were proposed to aid the identification of potential problems in the data or faulty preprocessing. NORM_anat_, NORM_func_, and AFO measures can be useful to evaluate functional normalization, anatomical normalization, and between modality coregistration success. Similarly, the relative severity of participant motion and other events that may cause outliers in the scan timeseries can be quantified using measures such as average of framewise displacement (MeanMotion), and the number or proportion of identified outlier scans (PVS). Measures evaluating the effective degrees of freedom of the BOLD signal timeseries after denoising (DOF), as well as its variability and intercorrelation (for example BOLDstd and GCOR), can also be useful to identify potential problems in the BOLD signal of individual participants before proceeding to statistical analyses. As other QC measures computed after preprocessing and denoising, outlier values in these measures may depend on the combination of most analytical steps that preceded it, so they do not directly suggest a potential source or cause of the identified problems. Finally, QC-FC correlations evaluate whether changes in the spatial correlation structure of the BOLD data covaried with participant-level quality control measures, such as the extent of participant motion, and the number or proportion of outlier scans, so they can be used as general measures of data quality to guide other data processing choices.

In this dataset these measures were used to evaluate the quality of the fMRI data and help guide our choices of denoising and exclusion procedures. Altogether, the QC pipeline and exclusion criteria adopted (Table 4) excluded 8% of the participants and minimized the presence of a variety of noise sources in the data as evaluated using a combination of visual and automated QC measures and procedures.

Many reasons may explain why bias persists after a successful preprocessing and adequate denoising, and these reasons create a multi(uni)verse of effective possibilities to counteract. Although relevant to the understanding of QC procedures, the evaluation of different processing pipelines was outside the scope of this paper and has been discussed in several seminal papers about preprocessing (Friston et al., 1996; Strother et al., 2004; Murphy et al., 2009; Chai et al., 2012; Hallquist et al., 2013; Power et al., 2014; Ciric et al., 2017) and denoising strategies (Churchill and Strother, 2013; Parkes et al., 2018; Maknojia et al., 2019; Tong et al., 2019; De Blasi et al., 2020; Golestani and Chen, 2022; for a review, see Caballero-Gaudes and Reynolds, 2017).

It is nevertheless important to note that not all measures that are used to evaluate the quality of the fMRI data in the context of QC procedures can or should be used to compare different preprocessing or denoising pipelines. In general, global or sample-level properties such as QC-FC %, characterizing between-subject QC-FC correlations, and FC mean ± SD, characterizing between-subjects variability in the shape of FC distributions, are meaningful measures that can be used to guide choices in preprocessing and denoising, and in particular to compare the relative success of different preprocessing pipelines. In contrast, many measures, such as BOLDstd, DOF, MeanGSchange, which are designed to provide useful contrasts when comparing different participants undergoing the same acquisition and analytical procedures, should be considered with extreme care in the context of comparing different analytical procedures or pipelines, as they provide only a very limited view of the overall quality of the data, with often contradictory results when interpreted as direct measures of data quality.

We encourage researchers to consider preprocessing and denoising strategies as an array of tools to use on their data, and rely on quality control measures described above to help guide and substantiate their choice of the best tools to use for each dataset. Indeed in our case, QC testing did suggest to evaluate alternative analytical approaches to attempt to improve the overall quality of the results. For example, there were two cases [sub-509 (S88) and sub-511 (S90)] in which anatomical normalization failed. This could have suggested that trying alternative normalization procedures customized to the dataset could have been tested. For example, normalization approaches using lesion-informed templates (which could have been relevant for site #5), age-specific normalization templates, or different normalization parameters could have led to overall better normalization performance for these two cases and perhaps others. Moreover, we did not perform STC to avoid introducing artificial heterogeneity between and within sites driven by differences in preprocessing pipelines. Our choice was based on a lack of information regarding slice timings for a portion (41.6%) of the data. But in a real-life context, we would have reached out to the research groups where the data originated trying to find said information. Similarly, we would have reached out to the site#5 to confirm that sub-518 (S97) and sub-519 (S98) functional data needed to be flipped rather than rotated. Also, the QC-FC 95% benchmark was not reached for PVS when considering data from all sites jointly (Figure 11, bottom row). That indicates that if we want to perform analyses jointly across all sites, we would need to correct site effects, as those potentially contain a mixture of noise sources together with perhaps other meaningful differences in sample demographics, but similarly other site homogenization approaches could be attempted to try to reduce or remove the residual QC-FC correlations across sites. In deciding the best course of action for the fmri-open-qc-rest collection, we faced a tradeoff between maximizing power (i.e., including as much data as possible) and prioritizing the optimal approach for the majority – but perhaps not the totality – of the data. Excluding a portion of runs (*n* = 11 out of 151 runs, corresponding to *n* = 11 out of 139 participants) resulted in an overall more lenient approach to the rest of the data and minimized the estimated residual bias driven by invalid scans, proportion of valid scans, and mean motion within each site independently and improved it across all sites jointly. Ultimately, the data and the research question motivating one’s own analysis will define what the “best” approach entails, potentially involving different analytical strategies. Whichever that is, we stress how reporting the rationale guiding preprocessing and denoising choices in a study and supporting those choices with reports describing the associated QC measures and procedures used, is a key element for results interpretation and reproducible science.

The proposed QC workflow, checklist, recommendations, and exclusion criteria are agnostic of the analytical software employed. While designed and discussed around the implementation in CONN, our recommendations generalize to data fully or partially analyzed (preprocessed and/or denoised) *via* other software packages including AFNI (Cox, 1996), SPM (Friston and Al, 2007), FSL (Jenkinson et al., 2012), FreeSurfer (Fischl, 2012), fMRIprep (Esteban et al., 2019), Tedana (DuPre et al., 2021), MRIQC (Esteban et al., 2017), pyfMRIQC (Williams and Lindner, 2020), and others. For example, NORM_anat_, NORM_func_, and AFO are measures diagnostic of preprocessed data quality, but they can be computed independently of the software or process that generated them. Furthermore, while the analytical details used to generate well-known metrics (framewise displacement, CompCor components, etc.) or methods (ICA, AROMA, CompCor) may vary across software packages, we expect that the recommendations provided in this manuscript should generalize beyond the specific measures used in the example presented in this manuscript. For example, we have no reasons to believe that the data exclusion based on the extreme departures of PVS relative to the sample’s distribution should be specific to the outlier threshold or motion estimation method that we used, rather they could generalize to alternative definitions of FD (Jenkinson et al., 2002; Power et al., 2012). In a similar fashion, considerations about visual QC could be expanded to apply to data inspected through MRI image viewers or visual plots generated with alternative methods.

The FMRI Open QC Project dataset (Taylor et al., 2022) combines information from multiple sites. The preprocessing, denoising, and QC steps discussed in this manuscript did not directly address the issue of data harmonization across sites (Friedman et al., 2006; Yu et al., 2018). Effective harmonization of features across sites would require a considerably richer array of information from the sampled participants in order to be able to differentiate among intersite differences that may carry meaningful information, such as those due to differences in age and health status of participants sampled in different sites or studies, from intersite differences that may be related to other factors of no interest, such as those introduced by specific acquisition details used in each study. Despite this, the quality control procedures described in this manuscript attempted to focus, whenever possible, on features of the entire dataset, treating *site* as one would normally treat different subject groups in a single-site study, except for QC-FC correlations, where we chose to focus only on intrasite analyses as otherwise the results would be naturally confounded by some of the very large differences in QC measures observed among sites. QC procedures in the context of multisite studies would benefit from an integrated approach to data homogenization and quality control, which is still an open area of research.

Most of the QC pipeline that we had described for resting state functional connectivity analysis is also suitable for task-based connectivity and task-based activation analyses. The QC workflow and exclusion criteria related to raw-level data visual inspection, preprocessed data visual and automated procedures (e.g., NORM_anat_, NORM_func_, AFO, and PVS) apply to (f)MRI data regardless of the final intended analysis goal. However the nature of the analysis (connectivity vs. activation) and of the behavioral/cognitive processes elicited during data acquisition (to rest or to perform an explicit task) carry distinct potential dangers on the final statistical analyses and require customized considerations. For example, motion is highly problematic for functional connectivity analysis, as it introduces biases reducing the accuracy of results, so it is thus usually more aggressively controlled for in the context of resting state analyses. In contrast, in task-activation studies, this is usually less of a concern as motion tends to simply reduce power (i.e., lowering statistical significance of the results) rather than introducing spurious results. Yet, activation analysis could suffer from a similar curse when motion artifacts are unbalanced between task conditions (e.g., larger subject motion during rest blocks compared to task blocks), so in the context of task-activation analyses QC measures that focus on the presence of task-correlated motion are often recommended. While the general QC workflow described in this manuscript can be equally used in the context of task-activation or other types of analyses, we would expect that the inclusion of additional QC measures focusing on analysis-specific features or sources of concern (e.g., quantifying the presence of task-correlated motion or other task-correlated noise sources in the context of task-activation analyses) would be necessary in order to better capture the suitability of the resulting data for those specific analyses.

Overall, the guidelines of our QC approach were to improve data quality and quantify residual nuisance effects. However, these guidelines were constrained by at least four limitations, which are the objective of open and active lines of work in the neuroimaging field. First, the field currently lacks a ground truth of what the BOLD signal is. It follows that quantifying the differences between the actual signal and the true signal was limited in its scope. Second, neural and non-neural signals are best thought of as a continuum rather than two ontological classes. Although regarded as a viable approach to minimize well-known bias, regressing out “non-neural” components might also have removed neural signals too (for example see Wang et al., 2021). Third, we applied similar processing to all data regardless of specific acquisition parameters, but it has been shown that non-harmonized MRI data could introduce spurious heterogeneity in FC estimates. However, potential sources of heterogeneity (e.g., inter-run, inter-participant, and inter-site variability; Greve et al., 2012) may be intertwined with true individual differences. Considering all available data, hence maximizing power and heterogeneity, may promote generalizability and reproducibility of neuroimaging results. Lastly, we defined exclusion criteria and cutoffs based on relative terms rather than absolute, which risks leading further away from a standardization of QC procedures. However, we argue that this shortcoming not only provides a necessary level of flexibility in view of the heterogeneity in acquisition details, sample characteristics, and experimental designs across different studies and fields, but also that it might effectively be overcome if QC procedures were to be consistently reported alongside FC results, however varied the QC strategies may be. Similarly to how distinct analytical approaches are regarded as equally valid in addressing the same research questions (Botvinik-Nezer et al., 2020), different QC pipelines could represent effective alternatives. As the description of the processing analytical details applied to fMRI data are considered necessary for interpretation and replicability purposes, likewise QC procedures are instrumental to results interpretation. Thus, QC reporting should become an integral part of neuroimaging studies.

## 6. Conclusion

In this study, we presented the CONN quality control pipeline for the visual and automated QC testing of resting state fMRI data for FC-MRI analysis, demonstrated on publicly available and heterogeneous data. We complemented knowledge and guidelines from the literature with additional automated QC strategies. Several, modular, and mutually non-exclusive procedures were included and emphasized how automated QC testing can help guide choices of preprocessing, denoising, and exclusion procedures. Overall, visual and automated QC were reciprocally informative, and their synergy was necessary for a sensitive evaluation of fMRI quality at all stages of the data life cycle. We hope this work contributes to the understanding, dissemination, and standardization of QC testing and QC reporting among peers and in scientific journals.

## Data availability statement

Publicly available datasets were analyzed in this study. This data can be found here: fMRI Open QC Project, https://osf.io/qaesm/files/osfstorage.

## Ethics statement

The studies involving human participants were reviewed and approved by fMRI Open QC Project. Written informed consent for participation was not required for this study in accordance with the national legislation and the institutional requirements.

## Author contributions

FM, AN-C, and SW-G contributed to the design of the study, data analysis, data interpretation, and manuscript writing. AN-C developed and maintains the CONN toolbox software. All authors revised and approved the submitted manuscript.

## Conflict of interest

The authors declare that the research was conducted in the absence of any commercial or financial relationships that could be construed as a potential conflict of interest.

## Publisher’s note

All claims expressed in this article are solely those of the authors and do not necessarily represent those of their affiliated organizations, or those of the publisher, the editors and the reviewers. Any product that may be evaluated in this article, or claim that may be made by its manufacturer, is not guaranteed or endorsed by the publisher.

## Footnotes

^1^ https://www.nitrc.org/projects/cpac/

## References

[ref1] Alfaro-Almagro F. Jenkinson M. Bangerter N. K. Andersson J. L. R. Griffanti L. Douaud G. . (2018). Image processing and quality control for the first 10,000 brain imaging datasets from UK biobank. NeuroImage 166, 400–424. doi: 10.1016/j.neuroimage.2017.10.034, PMID: 29079522 PMC5770339

[ref2] Andersson J. L. R. Hutton C. Ashburner J. Turner R. Friston K. (2001). Modeling geometric deformations in EPI time series. NeuroImage 13, 903–919. doi: 10.1006/nimg.2001.0746, PMID: 11304086

[ref3] Ashburner J. Friston K. J. (2005). Unified segmentation. NeuroImage 26, 839–851. doi: 10.1016/j.neuroimage.2005.02.018, PMID: 15955494

[ref4] Backhausen L. L. Herting M. M. Buse J. Roessner V. Smolka M. N. Vetter N. C. (2016). Quality control of structural MRI images applied using FreeSurfer—A hands-on workflow to rate motion artifacts. Front. Neurosci. 10:558. doi: 10.3389/fnins.2016.00558, PMID: 27999528 PMC5138230

[ref5] Behzadi Y. Restom K. Liau J. Liu T. T. (2007). A component based noise correction method (CompCor) for BOLD and perfusion based fMRI. NeuroImage 37, 90–101. doi: 10.1016/j.neuroimage.2007.04.042, PMID: 17560126 PMC2214855

[ref6] Benhajali Y. Badhwar A. Spiers H. Urchs S. Armoza J. Ong T. . (2020). A standardized protocol for efficient and reliable quality control of brain registration in functional MRI studies. Front. Neuroinform. 14:7. doi: 10.3389/fninf.2020.00007, PMID: 32180712 PMC7059806

[ref7] Bianciardi M. Fukunaga M. van Gelderen P. Horovitz S. G. de Zwart J. A. Shmueli K. . (2009). Sources of functional magnetic resonance imaging signal fluctuations in the human brain at rest: A 7 T study. Magn. Reson. Imaging 27, 1019–1029. doi: 10.1016/j.mri.2009.02.004, PMID: 19375260 PMC3512098

[ref8] Biswal B. B. Mennes M. Zuo X.-N. Gohel S. Kelly C. Smith S. M. . (2010). Toward discovery science of human brain function. Proc. Natl. Acad. Sci. 107, 4734–4739. doi: 10.1073/pnas.0911855107, PMID: 20176931 PMC2842060

[ref9] Botvinik-Nezer R. Holzmeister F. Camerer C. F. Dreber A. Huber J. Johannesson M. . (2020). Variability in the analysis of a single neuroimaging dataset by many teams. Nature 582, 84–88. doi: 10.1038/s41586-020-2314-9, PMID: 32483374 PMC7771346

[ref10] Caballero-Gaudes C. Reynolds R. C. (2017). Methods for cleaning the BOLD fMRI signal. NeuroImage 154, 128–149. doi: 10.1016/j.neuroimage.2016.12.018, PMID: 27956209 PMC5466511

[ref11] Calhoun V. D. Wager T. D. Krishnan A. Rosch K. S. Seymour K. E. Nebel M. B. . (2017). The impact of T1 versus EPI spatial normalization templates for fMRI data analyses. Hum. Brain Mapp. 38, 5331–5342. doi: 10.1002/hbm.23737, PMID: 28745021 PMC5565844

[ref12] Chai X. J. Castañón A. N. Öngür D. Whitfield-Gabrieli S. (2012). Anticorrelations in resting state networks without global signal regression. NeuroImage 59, 1420–1428. doi: 10.1016/j.neuroimage.2011.08.048, PMID: 21889994 PMC3230748

[ref13] Chou Y. Chang C. Remedios S. W. Butman J. A. Chan L. Pham D. L. (2022). Automated classification of resting-state fMRI ICA components using a deep Siamese network. Front. Neurosci. 16:768634. doi: 10.3389/fnins.2022.768634, PMID: 35368292 PMC8971556

[ref14] Churchill N. W. Strother S. C. (2013). PHYCAA+: An optimized, adaptive procedure for measuring and controlling physiological noise in BOLD fMRI. NeuroImage 82, 306–325. doi: 10.1016/j.neuroimage.2013.05.102, PMID: 23727534

[ref15] Ciric R. Wolf D. H. Power J. D. Roalf D. R. Baum G. L. Ruparel K. . (2017). Benchmarking of participant-level confound regression strategies for the control of motion artifact in studies of functional connectivity. NeuroImage 154, 174–187. doi: 10.1016/j.neuroimage.2017.03.020, PMID: 28302591 PMC5483393

[ref16] Cox R. W. (1996). AFNI: Software for analysis and visualization of functional magnetic resonance Neuroimages. Comput. Biomed. Res. 29, 162–173. doi: 10.1006/cbmr.1996.0014, PMID: 8812068

[ref17] Craddock C. Sikka S. Cheung B. Khanuja R. Ghosh S. S. Yan C. . (2013). Towards automated analysis of connectomes: The configurable pipeline for the analysis of connectomes (C-PAC). Front. Neuroinform. Conference Abstract: Neuroinformatics 7:42. doi: 10.3389/conf.fninf.2013.09.00042

[ref18] De Blasi B. Caciagli L. Storti S. F. Galovic M. Koepp M. Menegaz G. . (2020). Noise removal in resting-state and task fMRI: Functional connectivity and activation maps. J. Neural Eng. 17:046040. doi: 10.1088/1741-2552/aba5cc, PMID: 32663803

[ref19] Di Martino A. Yan C.-G. Li Q. Denio E. Castellanos F. X. Alaerts K. . (2013). The autism brain imaging data exchange: Towards a large-scale evaluation of the intrinsic brain architecture in autism. Mol. Psychiatry 19, 659–667. doi: 10.1038/mp.2013.78 23774715 PMC4162310

[ref20] DuPre E. Salo T. Ahmed Z. Bandettini P. Bottenhorn K. Caballero-Gaudes C. . (2021). TE-dependent analysis of multi-echo fMRI with tedana. J. Open Source Softw. 6:3669. doi: 10.21105/joss.03669, PMID: 36328274

[ref21] Esteban O. Birman D. Schaer M. Koyejo O. O. Poldrack R. A. Gorgolewski K. J. (2017). MRIQC: Advancing the automatic prediction of image quality in MRI from unseen sites. PLoS One 12:e0184661. doi: 10.1371/journal.pone.0184661, PMID: 28945803 PMC5612458

[ref22] Esteban O. Markiewicz C. J. Blair R. W. Moodie C. A. Isik A. I. Erramuzpe A. . (2019). fMRIPrep: A robust preprocessing pipeline for functional MRI. Nat. Methods 16, 111–116. doi: 10.1038/s41592-018-0235-4, PMID: 30532080 PMC6319393

[ref23] Fischl B. (2012). FreeSurfer. NeuroImage 62, 774–781. doi: 10.1016/j.neuroimage.2012.01.021, PMID: 22248573 PMC3685476

[ref24] Friedman L. Glover G. H. (2006). Report on a multicenter fMRI quality assurance protocol. J. Magn. Reson. Imaging 23, 827–839. doi: 10.1002/jmri.20583, PMID: 16649196

[ref25] Friedman L. Glover G. H. The FBIRN Consortium (2006). Reducing interscanner variability of activation in a multicenter fMRI study: Controlling for signal-to-fluctuation-noise-ratio (SFNR) differences. NeuroImage 33, 471–481. doi: 10.1016/j.neuroimage.2006.07.012, PMID: 16952468

[ref26] Friston K. J. Al E. (2007). Statistical parametric mapping: The analysis of functional brain images. London: Academic.

[ref27] Friston K. J. Williams S. Howard R. Frackowiak R. S. J. Turner R. (1996). Movement-related effects in fMRI time-series. Magn. Reson. Med. 35, 346–355. doi: 10.1002/mrm.1910350312, PMID: 8699946

[ref28] Glover G. H. Mueller B. A. Turner J. A. van Erp T. G. M. Liu T. T. Greve D. N. . (2012). Function biomedical informatics research network recommendations for prospective multicenter functional MRI studies. J. Magnet. Reson. Imaging 36, 39–54. doi: 10.1002/jmri.23572, PMID: 22314879 PMC3349791

[ref29] Golestani A. M. Chen J. J. (2022). Performance of temporal and spatial independent component analysis in identifying and removing low-frequency physiological and motion effects in resting-state fMRI. Front. Neurosci. 16:867243. doi: 10.3389/fnins.2022.867243, PMID: 35757543 PMC9226487

[ref30] Gorgolewski K. J. Auer T. Calhoun V. D. Craddock R. C. Das S. Duff E. P. . (2016). The brain imaging data structure, a format for organizing and describing outputs of neuroimaging experiments. Sci. Data 3:160044. doi: 10.1038/sdata.2016.44 27326542 PMC4978148

[ref31] Greve D. N. Brown G. G. Mueller B. A. Glover G. Liu T. T. (2012). A survey of the sources of noise in fMRI. Psychometrika 78, 396–416. doi: 10.1007/s11336-012-9294-0 25106392

[ref32] Griffanti L. Douaud G. Bijsterbosch J. Evangelisti S. Alfaro-Almagro F. Glasser M. F. . (2017). Hand classification of fMRI ICA noise components. NeuroImage 154, 188–205. doi: 10.1016/j.neuroimage.2016.12.036, PMID: 27989777 PMC5489418

[ref33] Hagler D. J. Hatton S. N. Cornejo M. D. Makowski C. Fair D. A. Dick A. S. . (2019). Image processing and analysis methods for the adolescent brain cognitive development study. NeuroImage 202:116091. doi: 10.1016/j.neuroimage.2019.116091, PMID: 31415884 PMC6981278

[ref34] Hallquist M. N. Hwang K. Luna B. (2013). The nuisance of nuisance regression: Spectral misspecification in a common approach to resting-state fMRI preprocessing reintroduces noise and obscures functional connectivity. NeuroImage 82, 208–225. doi: 10.1016/j.neuroimage.2013.05.116, PMID: 23747457 PMC3759585

[ref01] Henson R. N. A. Buechel C. Josephs O. Friston K. J. (1999). The slice- timing problem in event-related fMRI. NeuroImage 9, 1–125., PMID: 9918725

[ref35] Jenkinson M. Bannister P. Brady M. Smith S. (2002). Improved optimization for the robust and accurate linear registration and motion correction of brain images. NeuroImage 17, 825–841. doi: 10.1006/nimg.2002.1132, PMID: 12377157

[ref36] Jenkinson M. Beckmann C. F. Behrens T. E. J. Woolrich M. W. Smith S. M. (2012). FSL. NeuroImage 62, 782–790. doi: 10.1016/j.neuroimage.2011.09.015, PMID: 21979382

[ref37] Li X. Morgan P. S. Ashburner J. Smith J. Rorden C. (2016). The first step for neuroimaging data analysis: DICOM to NIfTI conversion. J. Neurosci. Methods 264, 47–56. doi: 10.1016/j.jneumeth.2016.03.001, PMID: 26945974

[ref38] Liu T. T. (2016). Noise contributions to the fMRI signal: An overview. NeuroImage 143, 141–151. doi: 10.1016/j.neuroimage.2016.09.008 27612646

[ref39] Liu T. T. Falahpour M. (2020). Vigilance effects in resting-state fMRI. Front. Neurosci. 14:321. doi: 10.3389/fnins.2020.00321, PMID: 32390792 PMC7190789

[ref40] Liu T. T. Glover G. H. Mueller B. A. Greve D. N. Rasmussen J. Voyvodic J. T. . (2015). “Quality assurance in functional MRI,” in fMRI: From nuclear spins to brain functions. Biological magnetic resonance. eds. Uludag K. Ugurbil K. Berliner L. (Boston, MA: Springer).

[ref41] Lu W. Dong K. Cui D. Jiao Q. Qiu J. (2019). Quality assurance of human functional magnetic resonance imaging: A literature review. Quant. Imaging Med. Surgery 9, 1147–1162. doi: 10.21037/qims.2019.04.18, PMID: 31367569 PMC6629553

[ref42] Maknojia S. Churchill N. W. Schweizer T. A. Graham S. J. (2019). Resting state fMRI: Going through the motions. Front. Neurosci. 13:825. doi: 10.3389/fnins.2019.00825, PMID: 31456656 PMC6700228

[ref43] Marcus D. S. Harms M. P. Snyder A. Z. Jenkinson M. Wilson J. A. Glasser M. F. . (2013). Human connectome project informatics: Quality control, database services, and data visualization. NeuroImage 80, 202–219. doi: 10.1016/j.neuroimage.2013.05.077, PMID: 23707591 PMC3845379

[ref44] Markiewicz C. J. Gorgolewski K. J. Feingold F. Blair R. Halchenko Y. O. Miller E. . (2021). The open neuro resource for sharing of neuroscience data. eLife 10, 1–17. doi: 10.7554/eLife.71774, PMID: 34658334 PMC8550750

[ref45] Murphy K. Birn R. M. Handwerker D. A. Jones T. B. Bandettini P. A. (2009). The impact of global signal regression on resting state correlations: Are anti-correlated networks introduced? NeuroImage 44, 893–905. doi: 10.1016/j.neuroimage.2008.09.036, PMID: 18976716 PMC2750906

[ref46] Nieto-Castanon A. (2020). Handbook of functional connectivity magnetic resonance imaging methods in CONN. Boston, MA: Hilbert Press. doi: 10.56441/hilbertpress.2207.6598

[ref47] Nieto-Castanon A. (2022). Preparing fMRI data for statistical analysis. ar xiv: 2210.13564 [q-bio]. Available at: https://arxiv.org/abs/2210.13564 (Accessed November 8, 2022).

[ref02] Nieto-Castanon A. Whitfield-Gabrieli S. (2022). CONN functional connectivity toolbox: RRID SCR_009550, release 22. Boston, MA: Hilbert Press. doi: 10.56441/hilbertpress.2246.5840, PMID:

[ref48] Parkes L. Fulcher B. Yücel M. Fornito A. (2018). An evaluation of the efficacy, reliability, and sensitivity of motion correction strategies for resting-state functional MRI. NeuroImage 171, 415–436. doi: 10.1016/j.neuroimage.2017.12.073, PMID: 29278773

[ref49] Power J. D. (2017). A simple but useful way to assess fMRI scan qualities. NeuroImage 154, 150–158. doi: 10.1016/j.neuroimage.2016.08.009, PMID: 27510328 PMC5296400

[ref50] Power J. D. Barnes K. A. Snyder A. Z. Schlaggar B. L. Petersen S. E. (2012). Spurious but systematic correlations in functional connectivity MRI networks arise from subject motion. NeuroImage 59, 2142–2154. doi: 10.1016/j.neuroimage.2011.10.018, PMID: 22019881 PMC3254728

[ref51] Power J. D. Mitra A. Laumann T. O. Snyder A. Z. Schlaggar B. L. Petersen S. E. (2014). Methods to detect, characterize, and remove motion artifact in resting state fMRI. NeuroImage 84, 320–341. doi: 10.1016/j.neuroimage.2013.08.048, PMID: 23994314 PMC3849338

[ref52] Raamana P. R. Theyers A. Selliah T. Bhati P. Arnott S. R. Hassel S. . (2020). Visual QC protocol for FreeSurfer cortical Parcellations from anatomical MRI. bioRxiv. doi: 10.1101/2020.09.07.286807

[ref53] Saad Z. S. Reynolds R. C. Jo H. J. Gotts S. J. Chen G. Martin A. . (2013). Correcting brain-wide correlation differences in resting-state FMRI. Brain Connect. 3, 339–352. doi: 10.1089/brain.2013.0156, PMID: 23705677 PMC3749702

[ref54] Sikka S.. Cheung B. Khanuja R. Ghosh S. Yan C. Li Q. Vogelstein J. Burns R. Colcombe S. Craddock C. Mennes M. Kelly C. Dimartino A. Castellanos F. Milham M. (2014). Towards automated analysis of connectomes: The configurable pipeline for the analysis of connectomes (C-PAC). The Configurable Pipeline for the Analysis of Connectomes (C-PAC). 5th INCF Congress of Neuroinformatics, Munich, Germany.

[ref55] Storelli L. Rocca M. A. Pantano P. Pagani E. De Stefano N. Tedeschi G. . (2019). MRI quality control for the Italian neuroimaging network initiative: Moving towards big data in multiple sclerosis. J. Neurol. 266, 2848–2858. doi: 10.1007/s00415-019-09509-4, PMID: 31422457

[ref56] Strother S. La Conte S. Kai Hansen L. Anderson J. Zhang J. Pulapura S. . (2004). Optimizing the fMRI data-processing pipeline using prediction and reproducibility performance metrics: I A preliminary group analysis. NeuroImage 23, S196–S207. doi: 10.1016/j.neuroimage.2004.07.022, PMID: 15501090

[ref57] Taylor P. Etzel J. Glen D. Reynolds R. Moraczewski D. Basavaraj A. (2022). FMRI open QC project. Available at: https://osf.io/qaesm/

[ref58] Tong Y. Hocke L. M. Frederick B. B. (2019). Low frequency systemic hemodynamic ‘noise’ in resting state BOLD fMRI: Characteristics, causes, implications, mitigation strategies, and applications. Front. Neurosci. 13:787. doi: 10.3389/fnins.2019.00787, PMID: 31474815 PMC6702789

[ref59] Wang P. Wang J. Michael A. Wang Z. Klugah-Brown B. Meng C. . (2021). White matter functional connectivity in resting-state fMRI: Robustness, reliability, and relationships to gray matter. Cereb. Cortex 32, 1547–1559. doi: 10.1093/cercor/bhab181, PMID: 34753176

[ref60] Whitfield-Gabrieli S. Nieto-Castanon A. (2012). Conn: A functional connectivity toolbox for correlated and anticorrelated brain networks. Brain Connect. 2, 125–141. doi: 10.1089/brain.2012.0073, PMID: 22642651

[ref61] Whitfield-Gabrieli S. Nieto-Castanon A. Ghosh S. (2011). Artifact detection tools (ART), Release version 7:11. Cambridge, MA: Artifact Detection Tools.

[ref62] Williams B. Lindner M. (2020). Pyf MRIqc: A software package for raw fMRI data quality assurance. J. Open Res. Softw. 8:23. doi: 10.5334/jors.280

[ref63] Yu M. Linn K. A. Cook P. A. Phillips M. L. McInnis M. Fava M. . (2018). Statistical harmonization corrects site effects in functional connectivity measurements from multi-site fMRI data. Hum. Brain Mapp. 39, 4213–4227. doi: 10.1002/hbm.24241, PMID: 29962049 PMC6179920

