# Supplementary material for: Functional connectivity MRI quality control procedures in CONN
Source: Front Neurosci. 2023 Mar 23;17:1092125. doi: 10.3389/fnins.2023.1092125 (PMC10076563; doi:10.3389/fnins.2023.1092125)
Supplement: Supplementary file 1 [file Data_Sheet_1.pdf]

Supplementary Material

Morfini, F., Whitfield-Gabrieli, S., Nieto-Castanon, A. (2023). Functional connectivity MRI quality control procedures in CONN. *Frontiers in Neuroscience*. 17:1092125. doi: 10.3389/fnins.2023.1092125

Supplementary Tables

Table S1. Anatomical MRI data information for each acquisition site.
Table S2. Field of view of anatomical MRI data of participants from site #5.
Table S3. List of excluded and uncertain data.

Supplementary Figures

Figure S1. Cross modality visual comparison with flipped and rotated functional data.
Figure S2. Example of raw-level artifacts.
Figure S3. Distribution of some automated QC derived from preprocessed data.
Figure S4. Distribution of automated QC measures representing tissue specific ROIs volumes.
Figure S5. Toy examples of functional connectivity distributions that could result from an overly or insufficiently aggressive denoising strategy.
Figure S6. Distribution of automated QC measures of the excluded runs, represented on the distribution of the full dataset.
Figure S7. Carpetplots of the excluded runs.

1 Supplementary Tables

Supplementary Table S1. Anatomical MRI data information for each acquisition site. *Note.* The information reported refers to all participants of each site, unless otherwise specified. 3D TFE, 3D turbo field echo; properties of a json file, key-value pairs included in the json files; MPRAGE, magnetization prepared rapid gradient echo; SENSE, sensitivity encoding; "/", information was not available; †, see supplementary Table S2.

	Site #1	Site #2	Site #3	Site #4	Site #5	Site #6	Site #7
N	20	20	16	23	20	20	20
Collection ID	sub-101 to 120	sub-201 to 220	sub-301 to 316	sub-401 to 423	sub-501 to 520	sub-601 to 620	sub-701 to 720
CONN ID	S1 to S20	S21 to S40	S41 to S56	S57 to S79	S80 to S99	S100 to S119	S120 to S139
MRI scanner	Philips Achieva	Philips Achieva	Philips Achieva	/	/	Siemens Magnetom	Siemens Verio
Data type	MPRAGE	MPRAGE	3D TFE	/	/	/	/
Flip angle [degrees]	8	8	8	/	/	8	8
Phase encoding direction	i	j-	i			/	i-
Parallel acquisition technique	SENSE	SENSE	SENSE	/	/	/	/
Voxel dimension [mm^3]	1x1x1x1 (13)<br>1x1x1x0 (7)	1x1x1x1	1x1x1x0	1x1x1x0	1x1x1x2 (15)<br>1x1x1x1 (5)	1x1x1x2	1x1x1x3
Field of view [slices]	256x200x256 (16)<br>256x180x256 (4)	160x256x256 (19)<br>160x288x288 (1)	256x182x256	256x200x256	†	256x256x256 (10)<br>256x256x176 (10)	192x256x256
Number of slices	200	160	182	/	/	/	/
Slices thickness	1	1	1.2	/	/	/	/
Parallel reduction factor in-plane	1	1	1.5	/	/	/	/
Parallel reduction factor out-of-plane	2	1.5	2.5	/	/	/	/
Acquisition duration [unspecified units]	488	449	103	/	/	/	/
Number of properties present in the json file	24	24	25	/	/	7	9

Supplementary Table S2. Field of view of anatomical MRI data of participants from site #5.
Note. [a] and [b] indicate that participant's anatomical data had identical voxel dimensions.

Collection ID	CONN ID	dimension1	dimension2	dimension3
sub-501	S80	160	188	189
sub-502	S81	160	192	175
sub-503	S82	160	193	156
sub-504	S83	160	194	159
sub-505	S84	160	195	200
sub-506	S85	160	196	200
sub-507	S86	160	197	165
sub-508	S87	160	202	158
sub-509	S88	160	206	143
sub-510	S89	160	209	150
sub-511	S90	160	212	198
sub-512	S91	160	216	200
sub-513	S92	160	218	200
sub-514	S93	160	255	200
sub-515 [a]	S94	160	256	256
sub-516 [a]	S95	160	256	256
sub-517 [b]	S96	256	256	150
sub-518 [b]	S97	256	256	150
sub-519 [b]	S98	256	256	150
sub-520 [b]	S99	256	256	150

Supplementary Table S3. List of excluded and uncertain data. *Note.* Extreme outliers are defined as runs with values either 3 times the interquartile range (IQR) below the 1st quartile (Q1) or above the 3rd quartile (Q3), and mild outliers were considered those values either 3 times interquartile range below the 1st quartile or above the 3rd quartile. We identified cases of raw-level data artifacts corrupted beyond repair as judged by the rater (criterion B), cases with extreme values as judged by a sample-specific Q3 + 3IQR or Q1-3IQR threshold criterion in $NORM_{anat}$ or AFO (criterion F), and in PVS, MeanMotion, GCOR, or DOF (criterion G). Refer to Table 4 in the main manuscript for further details about the definition of the exclusion criteria. AFO, anatomical to functional overlap; GCOR, global correlation; $NORM_{func}$, MNI-space template to functional overlap; $NORM_{anat}$, MNI-space template to anatomical overlap; PVS, proportion of valid scans.

Collection ID	CONN ID	Site	Excluded	Uncertain	Comments: visual inspection	Comments: automated QC measures
sub-106	6	site01		x	B (ringing patterns in anatomical)	
sub-107	7	site01		x	B (signal inhomogeneities zipper-like patterns in functional)<br>B (ringing patterns in anatomical)	
sub-109	9	site01		x	B (signal inhomogeneities, darker band in functional)	
sub-110	10	site01		x	B (ringing patterns in anatomical)	G (mild high outliers (GCOR = 0.05))
sub-111	11	site01		x	B (ringing patterns in anatomical)	G (extreme high outliers (GCOR = 0.05))
sub-113	13	site01		x		G (mild high outliers (GCOR = 0.04))
sub-114	14	site01		x	B (ringing patterns in anatomical)	
sub-115	15	site01		x	B (background noise or spill over in the APPA direction in functional)	
sub-118	18	site01	x (G_1)	x (G_2)		G_1 (extreme low outliers (PVS = 0.74))<br>G_2 (mild high outliers (InvalidScans = 41))
sub-119	19	site01		x	B (ringing patterns in anatomical)	G (mild low outliers (PVS = 0.83))
sub-203	23	site02		x	B (enlarged asymmetrical lateral ventricles)	
sub-205	25	site02		x		G (mild low outliers (PVS = 0.82, MeanMotion = 0.25))
sub-207	27	site02		x	B (ringing patterns in anatomical)	
sub-208	28	site02		x	B (Gibbs ringing patterns in anatomical)	
sub-209	29	site02		x	B (noise around the eyes, potentially outside of the brain in anatomical)	
sub-216	36	site02		x	B (unspecified artifact in anatomical)	
sub-217	37	site02		x	B (noise around the eyes, potentially outside of the brain in anatomical)	
sub-218	38	site02		x	B (Gibbs ringing patterns in anatomical)	G (mild high outliers (GCOR = 0.05))
sub-304	44	site03		x	B (signal dropout in functional)	
sub-305	45	site03		x	B (blurring in anatomical)	
sub-307	47	site03	x (G_1)	x (G_2)		G_1 (mild low outliers (PVS = 0.75))<br>G_2 (mild low outliers (MeanMotion = 0.25); mild high outliers (InvalidScans = 40))
sub-312	52	site03		x	B (unspecified noise visible on axial slices in functional)	
sub-314	54	site03		x	B (signal inhomogeneities zipper-like patterns in functional)	
sub-315	55	site03		x	B (signal inhomogeneities appearing as darker areas in functional, corresponding to undefined artifact or anatomical feature in anatomical)	
sub-316	56	site03		x		G (mild high outliers (MeanMotion = 0.27))
sub-405	61	site04	x (G_1)	x (B, G_2)	B (signal inhomogeneities, darker area localized in the interhemispheric fissure in functional)<br>B (unspecified brighter area (vessel) in the interhemispheric fissure in anatomical)	G_1 (extreme low outliers (PVS = 0.69))<br>G_2 (mild high outliers (InvalidScans = 38))

sub-406	62	site04		x	B (aliasing in anatomical)	
sub-407	63	site04		x	B (unspecified signal inhomogeneity in the right hemisphere, ringing-pattern, or band, or eyes spillover visible on axial slices in anatomical)	
sub-409	65	site04	x (B)		B (ghosting, spatial distortions, unspecified artifacts in functional)	
sub-410	66	site04		x	B (unspecified darker artifact visible on axial slices in functional)	
sub-411	67	site04		x	B (signal inhomogeneities zipper-like patterns in functional)	
sub-416	72	site04		x	B (unspecified background noise likely machine-related showing as small spheres 'floating' over the superior part of the head, apparently not affecting the brain in anatomical)	
sub-422	78	site04		x	B (brighter band cutting through superior-to-inferior regions and visible on coronal or sagittal slices in functional)	
sub-423	79	site04		x	B (unspecified signal inhomogeneity in the right hemisphere, ringing-pattern, or band, or eyes spillover visible on axial slices in anatomical)	
sub-501	80	site05		x	B (signal inhomogeneities appearing as bands bilaterally visible in coronal slices, possibly pulsation artifact in anatomical)	
sub-502	81	site05		x	B (signal inhomogeneities appearing as bands bilaterally visible on sagittal slices, possibly pulsation artifact in anatomical)<br>B (background noise in inferior slices in functional)	
sub-503	82	site05		x	B (background noise in inferior slices in functional)	
sub-507	86	site05		x		G (mild low outliers (PVS = 0.76); mild high outliers (InvalidScans = 35))
sub-508	87	site05		x		G (mild high outliers (GCOR = 0.04))
sub-509	88	site05	x (F)	x (B, G)	B (enlarged asymmetrical lateral ventricles)<br>B (signal inhomogeneities appearing as bands bilaterally visible in coronal slices, possibly pulsation artifact in anatomical)<br>B (signal inhomogeneities, darker area reaching from the cortex to subcortical areas, possibly suggesting past surgery or foreign body in functional and anatomical)<br>B (bands bilaterally visible in coronal slices in anatomical data)	F (extreme low outliers (AFO = 0.08; $NORM_{anat}$ = 0.46))<br>G (mild low outliers (PVS = 0.78); mild high outliers (InvalidScans = 32))
sub-510	89	site05		x	B (ghosting and background noise in functional)<br>B (signal inhomogeneities, darker area reaching from the cortex to subcortical areas, possibly suggesting past surgery or foreign body in anatomical)	
sub-511	90	site05	x (F)	x (G)		F (extreme low outliers ($NORM_{anat}$ = 0.60); mild low outliers: (AFO = 0.51))<br>G (mild low outliers (GCOR = 0.05))
sub-512	91	site05		x	B (signal inhomogeneities, darker areas in the frontal areas in functional)<br>B (unspecified darker area in the right frontal region in anatomical)	G (mild high outliers (GCOR = 0.04))
sub-514	93	site05		x	B (light ringing in anatomical)	
sub-515	94	site05		x	B (individual anatomical difference in left cerebellum in anatomical)	
sub-516	95	site05		x	B (signal inhomogeneities, darker areas in the frontal areas in functional)	

sub-517	96	site05		x	B (signal inhomogeneities, darker areas in the frontal areas in functional)<br>B (signal inhomogeneities, darker area reaching from the cortex to subcortical areas, possibly suggesting past surgery or foreign body in functional and anatomical)	
sub-518	97	site05		x	B (wrong orientation of functional)<br>B (signal inhomogeneities, darker areas in lateral area in functional)	
sub-519	98	site05	x (G_1)	x (B, G_2)	B (wrong orientation of functional)<br>B (aliasing or motion-related repetitive patterns affecting all the brain in anatomical)	G_1 (extreme low outliers (PVS = 0.54))<br>G_2 (extreme high outliers (InvalidScans = 66))
sub-601	100	site06		x		G (mild high outliers (InvalidScans = 42))
sub-602	101	site06		x	B (light unspecified ghosting, aliasing, or background noise in functional)	
sub-606	105	site06		x	B (light unspecified ghosting, aliasing, or background noise in functional)	
sub-607	106	site06		x	B (light unspecified ghosting, aliasing, or background noise in functional)	G (extreme high outliers (InvalidScans = 50))
sub-608	107	site06		x	B (light unspecified ghosting, aliasing, or background noise in functional)	
sub-612	111	site06		x	B (light unspecified ghosting, aliasing, or background noise in functional)	
sub-614	113	site06		x	B (light unspecified ghosting, aliasing, or background noise in functional both runs)	
sub-615	114	site06		x	B (light unspecified ghosting, aliasing, or background noise in functional both runs)	
sub-616	115	site06		x	B (light unspecified ghosting, aliasing, or background noise in functional both runs)	
sub-620	119	site06		x		G (mild high outliers (InvalidScans = 33))
sub-701	120	site07		x	B (darker areas localized near subcortical regions of unspecified nature in functional)	
sub-703	122	site07	x (G_1)	x (B, G_2)	B (ringing, spillover patterns in anatomical)	G_1 (extreme low outliers (PVS = 0.59))<br>G_2 (extreme high outliers (InvalidScans = 81))
sub-706	125	site07	x (G_1)	x (G_2)		G_1 (extreme low outliers (PVS = 0.67))<br>G_2 (extreme high outliers (InvalidScans = 66); mild high outliers (MeanMotion = 0.24))
sub-707	126	site07		x	B (darker localized area of unspecified nature in functional)	
sub-708	127	site07	x (G_1)	x (G_2)		G_1 (extreme low outliers (PVS = 0.69))<br>G_2 (extreme high outliers (InvalidScans = 61); mild high outliers (MeanMotion = 0.28))
sub-710	129	site07		x		F (mild low outliers ($NORM_{anat}$ = 0.71)
sub-713	132	site07		x		G (mild low outliers (PVS = 0.83); mild high outliers (InvalidScans = 34))
sub-714	133	site07	x (G_1)	x (B, G_2)	B (ringing patterns in anatomical)	G_1 (extreme low outliers (PVS = 0.45))<br>G_2 (extreme high outliers (InvalidScans = 108))
sub-715	134	site07		x		G (mild high outliers (MeanMotion = 0.24))
sub-716	135	site07		x	B (darker localized area of unspecified nature in functional)	F (mild low outliers ($NORM_{anat}$ = 0.70)
sub-717	136	site07		x	B (ghosting, aliasing, or background noise in functional)	
sub-719	138	site07		x	B (anatomical difference in lateral ventricle showing unspecified brighter area in anatomical)	

2 Supplementary Figures

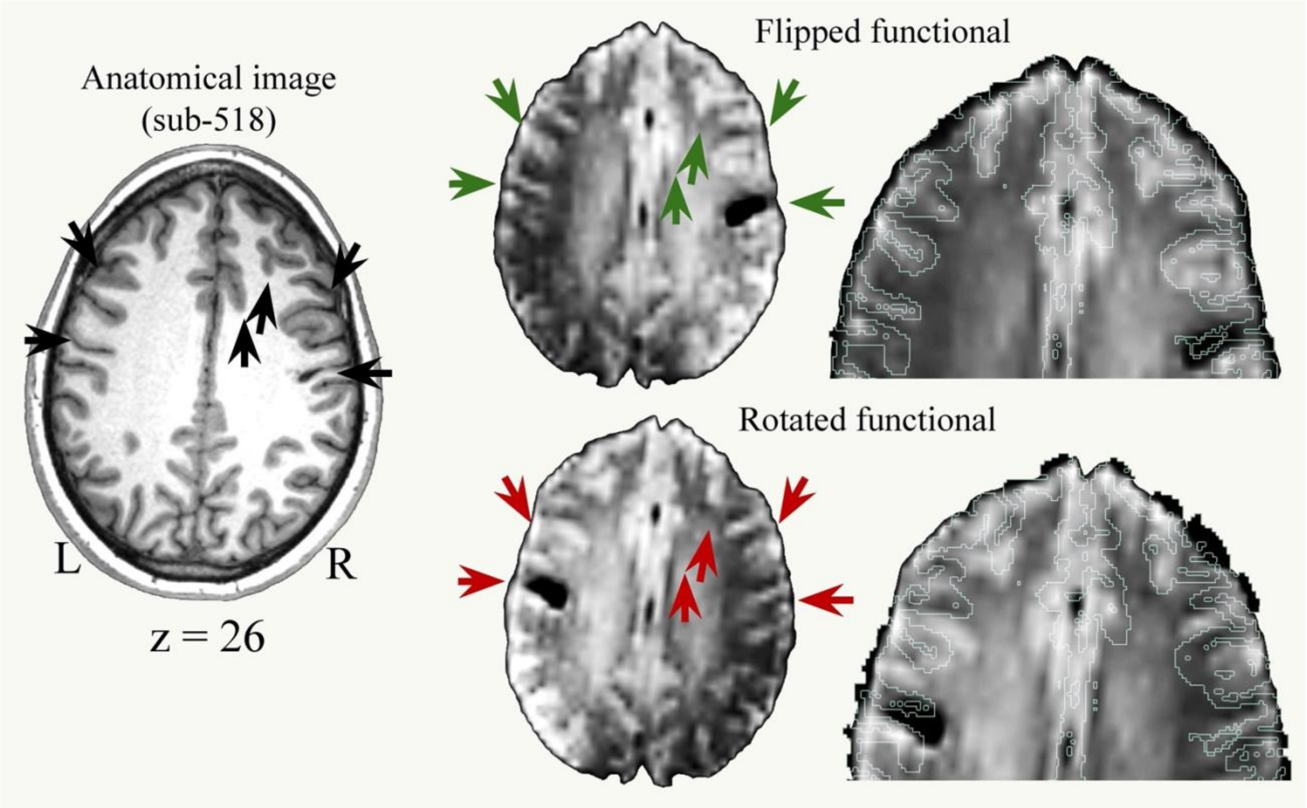

Supplementary Figure S1. Cross modality visual comparison with flipped and rotated functional data. Data from sub-519 (S97) was anatomical boundaries (blue outlines) and either flipped (left) or rotated (right) functional data. The same brain slice (subject-space z = 26) is rendered in both cases. Arrows indicated some exemplar areas of where anatomical matched better with flipped rather than rotated functional image. For visualization purposes, functional data was realigned, co-registered directly to the anatomical images without resampling, and not normalized nor smoothed.

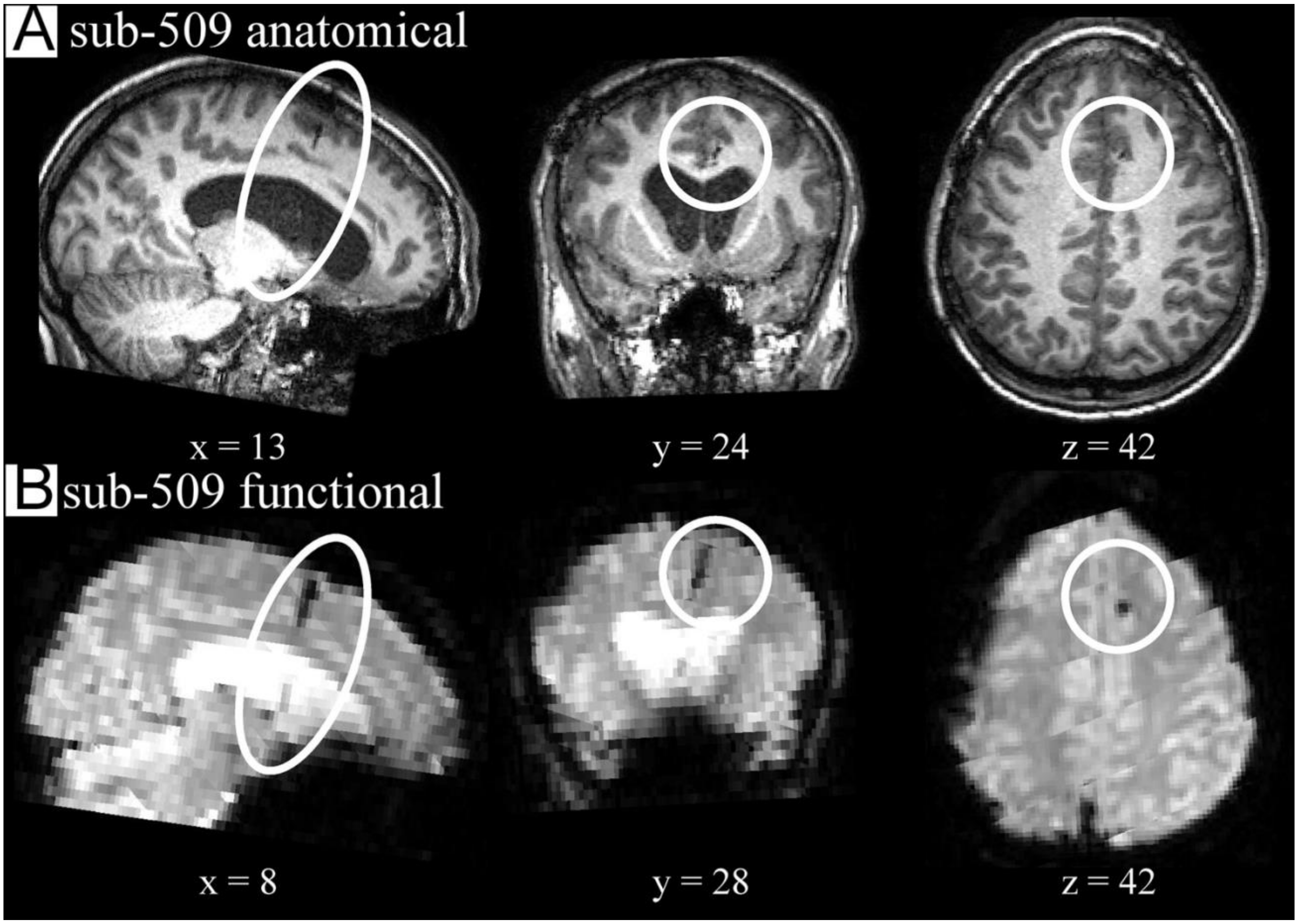

Supplementary Figure S2. Example of raw-level artifacts. Raw-level anatomical (**A**) and functional (**B**) data sub-509 (S88) showing correspondence between a potential tissue lesion or foreign body in the anatomical with sharp intensity differences in the functional data.

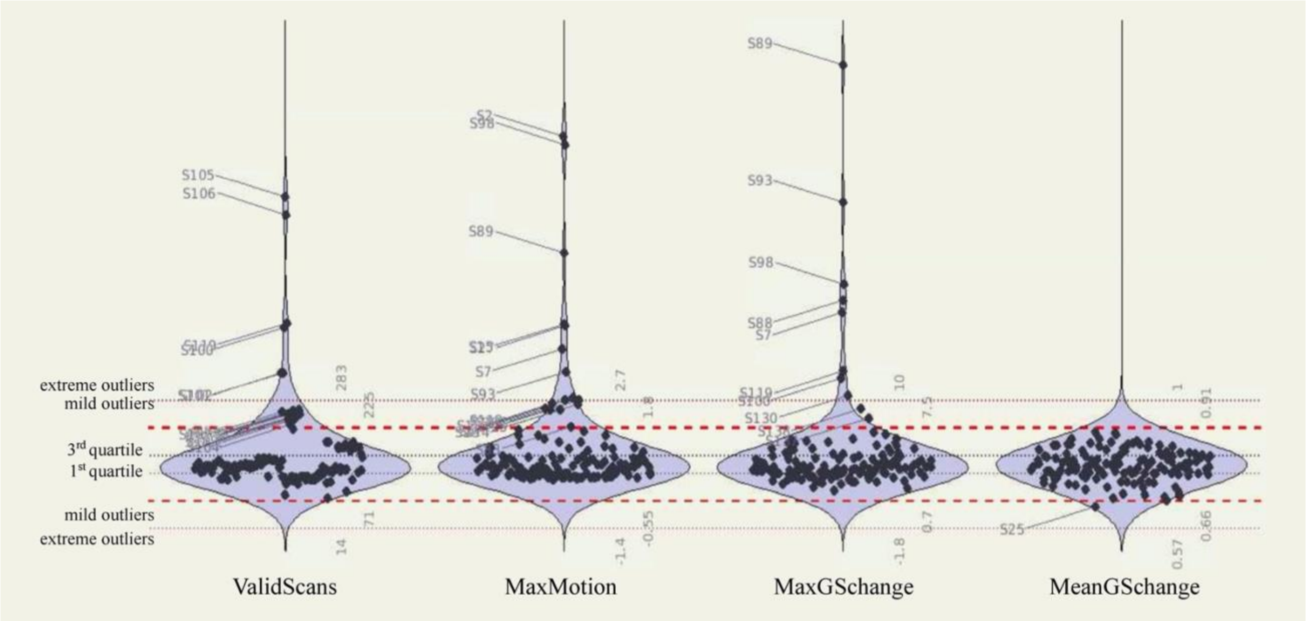

Supplementary Figure S3. Distribution of some automated QC derived from preprocessed data. Automated QC measures calculated from all data during or after preprocessing (n = 139 anatomical images and n = 151 functional runs).

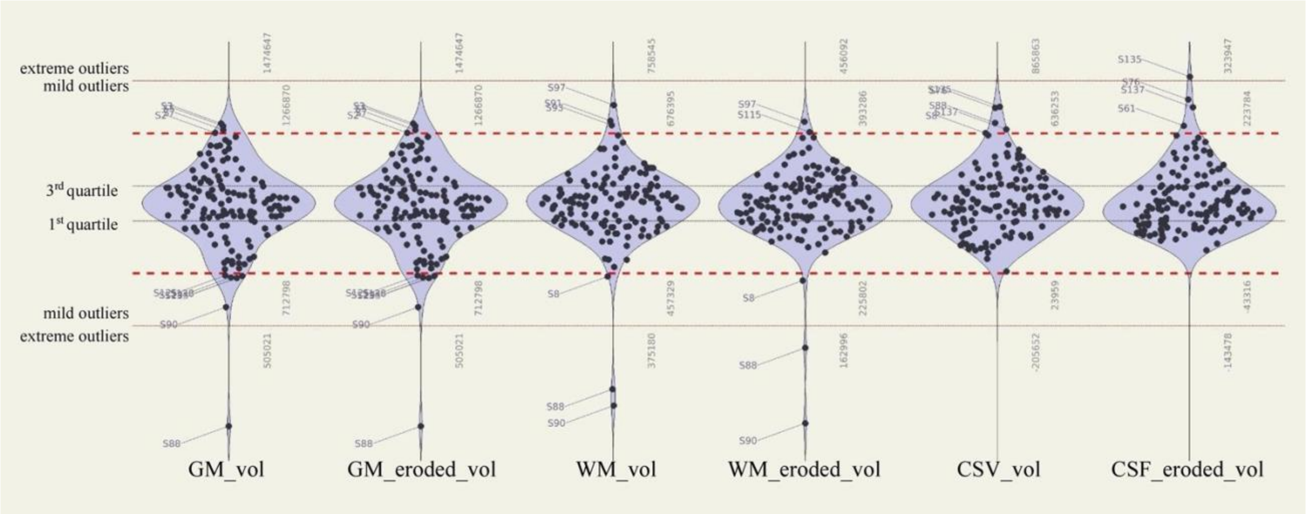

Supplementary Figure S4. Distribution of automated QC measures representing tissue specific ROIs volumes. Distribution of tissue volumes estimated from gray matter (GM), white matter (WM) and cerebrospinal fluid (CSF) generated during preprocessing and denoising (n = 138).

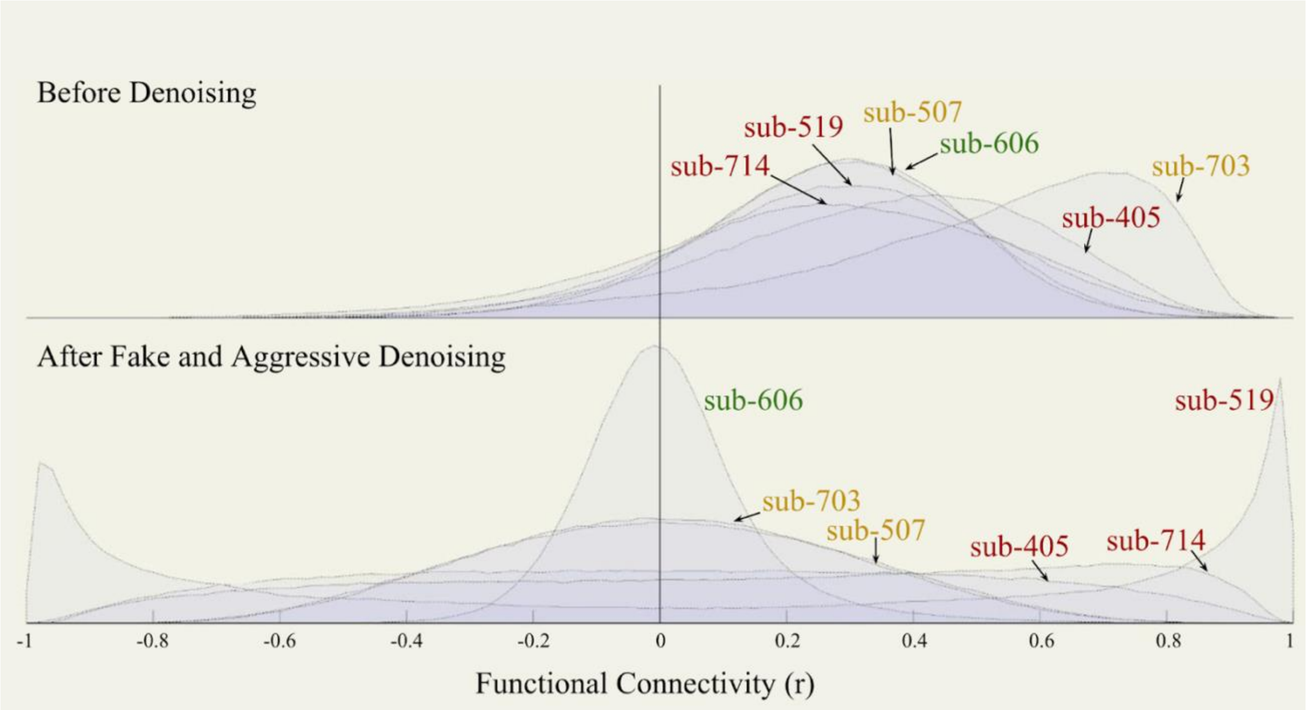

Supplementary Figure S5. Toy examples of functional connectivity distributions that could result from an overly or insufficiently aggressive denoising strategy. These data were denoised with an unreasonably overly aggressive approach with the only intent to create a visual representation of the problematic extreme scenarios discussed in the main manuscript (section 3.3.3). Density distributions of within-run FC strengths (r coefficients) between all pairs among 1,000 randomly selected voxels from n=6 functional runs are depicted before (top) and after denoising (bottom). The participant IDs are color coded based on their visual appearance after denoising as problematic (red), uncertain (yellow), or optimal (green) cases. After this toy denoising approach, sub-519 (S98) has a split/bimodal FC distribution, sub-714 (S133) and sub-405 (S61) present with flat distributions, whereas sub-703 (S122) and sub-507 (S86) have what could be considered borderline flat distributions which are to be interpreted with care. Lastly, sub-606 (S105) has an ideal FC distribution after denoising and it is used here as a reference sample. Its FC distribution is mostly symmetrical, centered around r=0, with small but noticeable tails, and with a slightly longer positive tail (i.e., note how the positive tail around r = 0.4 is slightly higher compared to the negative tail around r = -0.4).

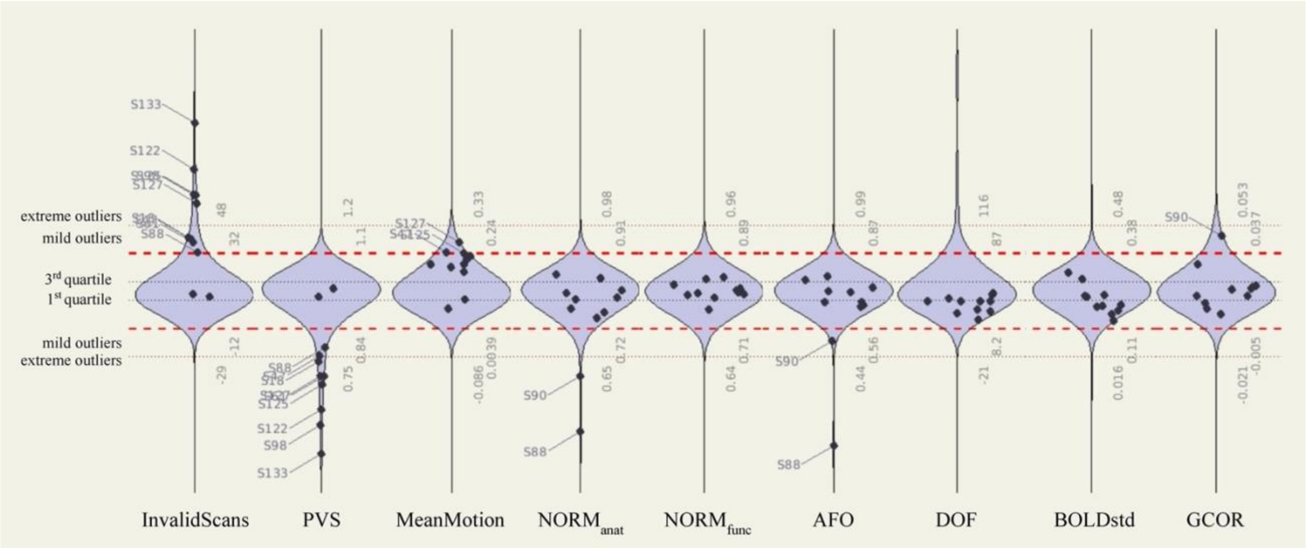

Supplementary Figure S6. Distribution of automated QC measures of the excluded runs, represented on the distribution of the full dataset. The distribution of the automated QC measures was calculated based on data of the full dataset (n = 139). Shown in the distributions, are the values corresponding to only the participants which were excluded throughout QC testing (n = 11).

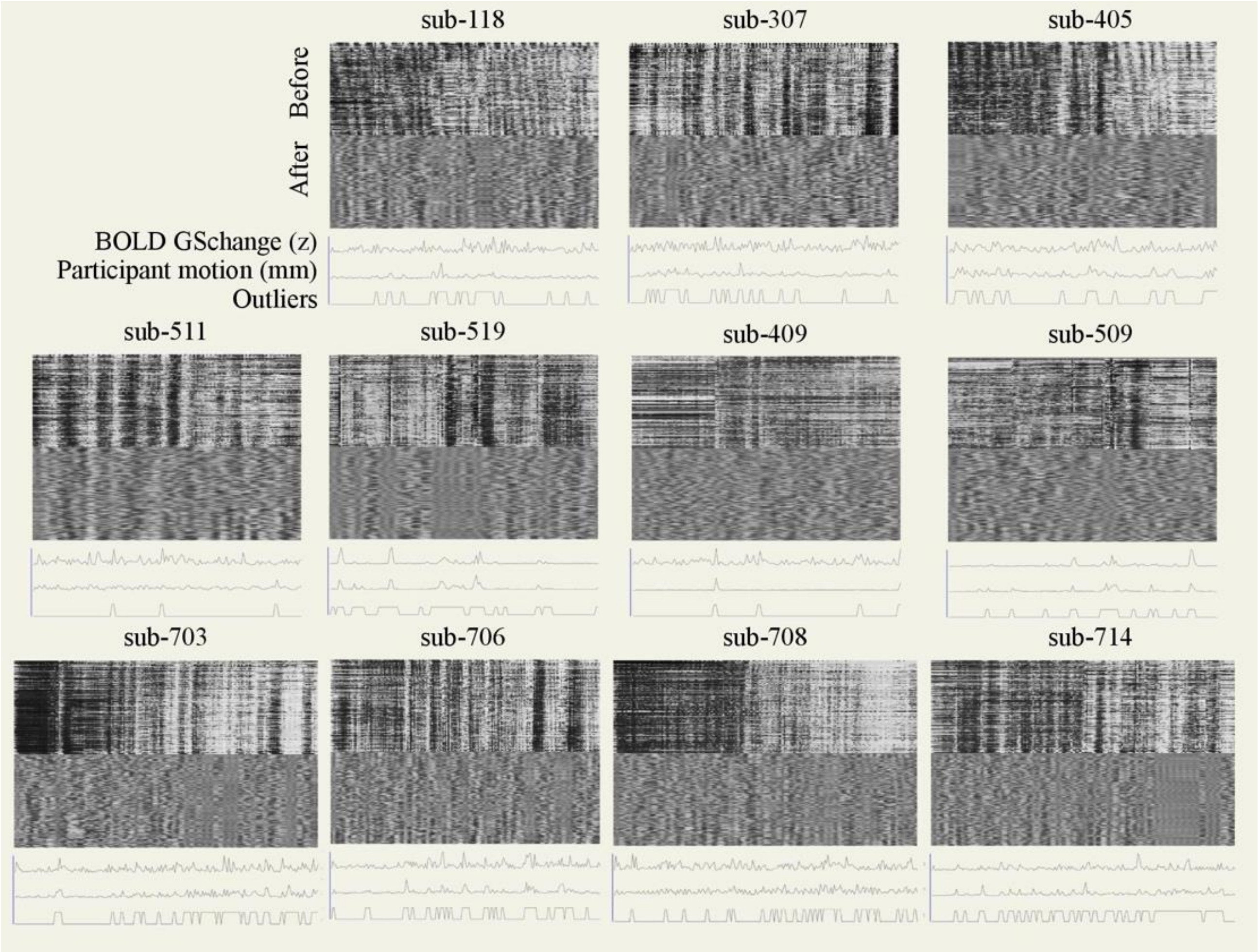

Supplementary Figure S7. Carpetplots of the excluded runs. Carpetplots of functional timeseries before (top) and after (bottom) denoising, overlaid on top of the GSchange timeseries (z score), estimated participant motion (mm), and outlier volumes identified. Here, we report the plots of the runs which were excluded throughout QC testing (n = 11) for any reason: sub-118 (S18), sub-307 (S47), and sub-405 (S61) for low PVS values; sub-511 (S65) for extreme raw-level data ghosting and aliasing; sub-519 (S88) and sub-409 (S90) for anatomical normalization failure; and sub-509 (S98), sub-703 (S122), sub-706 (S125), sub-708 (S127), sub-714 (S133) for low PVS values. BOLD, blood oxygen level dependent; GS, global signal.
